# Effectiveness of Exergame Intervention on Walking in Older Adults: A Systematic Review and Meta-Analysis of Randomized Controlled Trials

**DOI:** 10.1093/ptj/pzab152

**Published:** 2021-06-21

**Authors:** Maarit Janhunen, Vera Karner, Niina Katajapuu, Oona Niiranen, Jaakko Immonen, Juha Karvanen, Ari Heinonen, Eeva Aartolahti

**Affiliations:** Faculty of Sport and Health Sciences, University of Jyväskylä, Jyväskylä, Finland; Physiotherapy, University of Applied Sciences for Health Professions Upper Austria, Linz, Austria; Faculty of Sport and Health Sciences, University of Jyväskylä, Jyväskylä, Finland; Health and Well-Being, Turku University of Applied Sciences, Turku, Finland; Health and Well-Being, Turku University of Applied Sciences, Turku, Finland; Faculty of Sport and Health Sciences, University of Jyväskylä, Jyväskylä, Finland; Faculty of Mathematics and Science, University of Jyväskylä, Jyväskylä, Finland; Faculty of Sport and Health Sciences, University of Jyväskylä, Jyväskylä, Finland; Faculty of Sport and Health Sciences, University of Jyväskylä, Jyväskylä, Finland

**Keywords:** Aged, Exercise Therapy, Exergames, Rehabilitation, Walking

## Abstract

**Objective:**

The objective of this review was to systematically evaluate the effectiveness of exergaming on walking in older adults. In addition, the aim was to investigate the relationship between the exergaming effect and age, baseline walking performance, exercise traits, technology used, and the risk of bias.

**Methods:**

A literature search was carried out in the databases MEDLINE, CINAHL, CENTRAL, EMBASE, WoS, PsycInfo, and PEDro up to January 10, 2020. Studies with a randomized controlled trial design, people ≥60 years of age without neurological disorders, comparison group with other exercise or no exercise, and walking-related outcomes were included. Cochrane RoB2, meta-analysis, meta-regression, and Grading of Recommendations, Assessment, Development and Evaluation were used to estimate quality, treatment effect, covariates’ effect, and the certainty of evidence, respectively.

**Results:**

In the studies included (n = 66), the overall risk of bias was low (n = 2), unclear (n = 48), or high (n = 16). Compared with comparison groups, exergaming interventions were more effective for walking improvements (standardized mean difference = −0.21; 95% CI = −0.36 to −0.06; 3102 participants, 58 studies; moderate-quality evidence) and more or equally effective (standardized mean difference = −0.32; 95% CI = −0.64 to 0.00; 1028 participants, 13 studies; low-quality evidence) after nonexergaming follow-up. The strongest effect for covariates was observed with the type of comparison group, explaining 18.6% of the variance.

**Conclusion:**

For older adults without neurological disorders, exergame-based training improved walking, and improvements were maintained at follow-up. Greater benefits were observed when exergaming groups were compared with inactive comparison groups. To strengthen the evidence, further randomized controlled trials on the effectiveness of gamified exercise intervention are needed.

**Impact:**

Exergaming has an effect equivalent to other types of exercising on improving walking in older adults. Physical therapists and other rehabilitation professionals may consider exergaming as a promising form of exercise in this age group.

## Introduction

Walking is the leading form of mobility for older adults. It is one of the main areas of physical functioning that enables physical activity and independence,^1^ social participation,^2^ and good quality of life^3^ in older adults. Adequate physical activity that includes walking lowers the risk of major mobility disability among older adults.^4^^,^^5^ Evidence demonstrates that physical activity and exercise reduce the risk of age-related loss of physical functioning^6^ and multicomponent training, including strength and balance training, improves or maintains walking in older adults.^7^^,^^8^ One novel multicomponent training method that may be used to enhance walking is exercise games, known as exergames.

Exergames, also considered serious games, are computer-based video games that can be used for nonrecreational purposes, such as for physical rehabilitation targeting to correct and to restore musculoskeletal functions.^9^ Exergaming requires physical performance from the player, as the technology used in gaming system tracks the player’s physical movements to control the game, immersing the player in the game. In rehabilitation, exergaming may be targeted to enhance the physical functioning, such as walking performance,^10^ of different patient groups,^11^ and it may be carried out in a variety of settings, such as in unsupervised conditions.^12^^,^^13^ Exercising with exergames has been shown to be engaging^14^ and enjoyable^15^ among older adults and thus may increase training volume and contribute to the effectiveness of physical rehabilitation.

The scientific interest in exergaming as a potential rehabilitation method in older adults has grown with the increase of digitalization in physical rehabilitation.^9^ Evidence shows that exergaming is effective in improving walking in older adults with neurologic disorders.^16^ In addition, a positive effect on walking has been observed in older adults with no specific pathologies, but the evidence is based on pooled outcomes of physical performance^10^^,^^17^ or a variety of study designs, including non-randomized controlled trials (RCTs).^18^ To the best of our knowledge, no previous meta-analysis of RCTs has solely focused on walking in older adults without neurological disorders. Therefore, the aim of this meta-analysis was to summarize studies with an RCT design investigating the effectiveness of exergame-based intervention on walking in older adults without neurological disorders and to implement meta-regression to account for a risk of bias and heterogeneity in studies. The following questions were addressed: (1) Are exergame-based interventions more effective than comparison group interventions on walking in older adults? (2) Does participants’ age, baseline walking performance, duration of intervention, setting of intervention, number of sessions per week, duration of single session, type of comparison group, technology used, or risk of bias explain the effectiveness of exergame-based training on walking?

## Methods

This systematic review and meta-analysis of RCTs was registered prospectively in the International Prospective Register of Systematic Reviews (https://www.crd.york.ac.uk/prospero/display_record.php?ID=CRD42020148701) before completion of formal screening of search results against eligibility criteria and starting the data extraction. The reporting corresponds to the guidelines of Preferred Reporting Items for Systematic Reviews and Meta-Analyses.^19^

To study the effectiveness of computer-based exercise interventions on walking, only RCTs were included in the review. The eligibility criteria followed the PICO framework; participants were 60 years or older (P), experimental intervention was carried out with exergames that demanded physical movements (I), the comparison group had a different type of exercising protocol (active control) or no exercise protocol (inactive control) (C), and the study reported validated and standardized outcomes measuring walking (O). Theses and conference proceeding abstracts, studies focusing on patients with neurological disorders (such as stroke, Parkinson’s disease, or multiple sclerosis), studies using accelerometers or actual locations in the intervention to encourage and to record physical activity (eg, pedometers, Pokémon Go), and studies of exergames driven by player’s eyes, head, or fine physical movements were excluded from the review. No language or publication date restrictions were applied.

### Data Sources and Searches

RCTs were identified by searching 7 electronic databases in January 2019 and January 2020 (updated search) covering the earliest available date until January 10, 2020. The databases were US National Library of Medicine (MEDLINE, 1946 to present), Cumulative Index to Nursing and Allied Health Literature (CINAHL, 1981 to present), Cochrane Central Register of Controlled Trials (CENTRAL, 1991 to present), Comprehensive Biomedical Literature Database (EMBASE, 1947 to present), Web of Science (WoS, 1945 to present), Behavioral and Social Science Research (PsycInfo, 1887 to present), and Physiotherapy Evidence Database (PEDro, 1929 to present). In addition, research article publications were monitored from the review’s topic.

The key terms used to identify studies in the electronic search were exergame, exercise, and RCT. Synonyms, MeSH, and related terms of key terms (eg, video games for exergame, physical rehabilitation for exercise, and clinical trial for RCT) and RCT filters (MEDLINE [Ovid], Embase and CINAHL [Ebsco],^20^ Web of Science^21^) were combined by using the Boolean operators “OR” and “AND.” A full electronic search strategy in the MEDLINE database is presented in Supplementary Material A.

### Study Selection

References of identified studies were imported to the screening and data extraction tool^22^ in which duplicates were removed from the search results. Authors (M.J., V.K., N.K., O.N., and E.A.) in pairs independently assessed study titles and abstracts by applying the eligibility criteria. Research reports were collected for studies that were carried forward to the full-text screening. The pairs of authors independently reapplied eligibility criteria to full texts and reported reasons for the exclusion of ineligible studies. In the screening, disagreement between the pairs of authors was resolved by discussion between them or by the third author (M.J., N.K., or E.A.). All eligible RCTs were included in the systematic review and, if applicable, in the meta-analysis.

### Data Extraction and Quality Assessment

A customized format to report participants, interventions, and outcomes of studies included in the data extraction was created (M.J.) (Suppl. Material A), and the Cochrane Risk of Bias 2 tool^23^ was used in the quality assessment of individual studies at the outcome level. Before starting the data extraction and quality assessment, joint practice among the team of reviewing authors was conducted to ensure uniform data extraction and risk of bias evaluation. Two authors (M.J. and either V.K., N.K, O.N., or E.A.) performed the data extraction and risk of bias evaluation independently. When necessary, disagreements between the 2 authors in the evaluations were resolved by discussion between them or by a third independent author (E.A.).

Eighteen original researchers were contacted no more than 3 times via email and ResearchGate because of inadequate participant (2) or outcome data (14) and ambiguities (2) found in the published papers. Eleven of them responded and provided participant and outcome data (1 and 10, respectively) (Suppl. Material A). Journal articles, trial protocols, and trial registry records were used in risk of bias assessments, with a focus on the effect of assignment to intervention (the intention-to-treat effect). All studies, regardless of risk of bias judgement, were included in the narrative synthesis and the meta-analysis.

### Data Synthesis and Analysis

Improvement in walking was the primary measure of treatment effect. To assess the treatment effect after intervention and when available after follow-up periods, the meta-analysis was performed in R^24^ with the metafor package^25^ using a random effects model and restricted maximum-likelihood estimation. In the quantitative synthesis, post-intervention and follow-up mean and SD values of continuous outcomes were used to calculate standardized mean differences (SMD, Hedges’ *g*) and 95% CIs between groups. The effect size was considered small (*g* = 0.2), medium (*g* = 0.5), or large (*g* = 0.8).^26^

For studies in which the mean and SD were not reported (n = 7), SMDs were calculated according to recommendations^27^ (Suppl. Material A). When post-intervention values were not available or the study had subgroups that did not match the review’s inclusion criteria, changes from baseline values and a subset of data were used in the meta-analysis and the meta-regression. For studies with multiple comparator groups, the dependency structure of the SMDs was taken into account in the variance–covariance matrix.^28^ The Grading of Recommendations, Assessment, Development and Evaluation^29^ was used to rate the certainty of evidence in the meta-analysis.

Synthesis was structured around the content of interventions and outcomes, the latter being prioritized according to incidence level, validity, and reliability to define a direction of the value (lower/higher is better) and to combine results from studies in the analysis (Suppl. Material A). Heterogeneity was explored with the *Q* and *I*^2^ statistic and assessed from forest and funnel plots.

#### Risk of Bias Across Studies

Bias caused by selective publication within studies was evaluated by assessing the funnel plot of the trial mean differences for asymmetry.^30^ The bias caused by selective reporting within studies was evaluated from the risk of bias assessment.

#### Meta-Regression

Meta-regression was conducted to assess high risk of bias and sources of heterogeneity between studies. The covariates included in the meta-regression were participants’ mean age and level of walking performance prior intervention, duration and setting (unsupervised) of intervention, number of sessions per week, session duration, type of comparison group (active or inactive), technology used (made for physical rehabilitation purposes), and risk of bias domains (high risk). Participants’ walking performance prior to intervention was evaluated from their baseline Timed Up & Go (TUG) test results. A separate meta-regression model was fitted for each covariate. In addition, a model including all covariates simultaneously was fitted. Heterogeneity accounted for by the covariates was measured using (pseudo) R^2^.^31^

#### Multiple Imputation

To avoid excluding studies from the meta-regression, multiple imputation was applied to the study-level covariates that were not measured in some of the studies. These variables included the TUG test, walking speed (calculated from a 2-minute walking test or from a 6-minute walking test), the number of sessions per week, and the session duration. The imputation model for the TUG test was a log-linear model, where the logarithm of the TUG test result was explained by participants’ mean age and the logarithm of the walking speed. The roles of the TUG test and the walking speed were exchanged when the walking speed was imputed. Multiple imputation by chained equations^32^ and the R package mice^33^ were used.

### Role of the Funding Source

The funders played no role in the design, conduct, or reporting of this study.

## Results

### Study Selection and Characteristics

**Figure 1:**
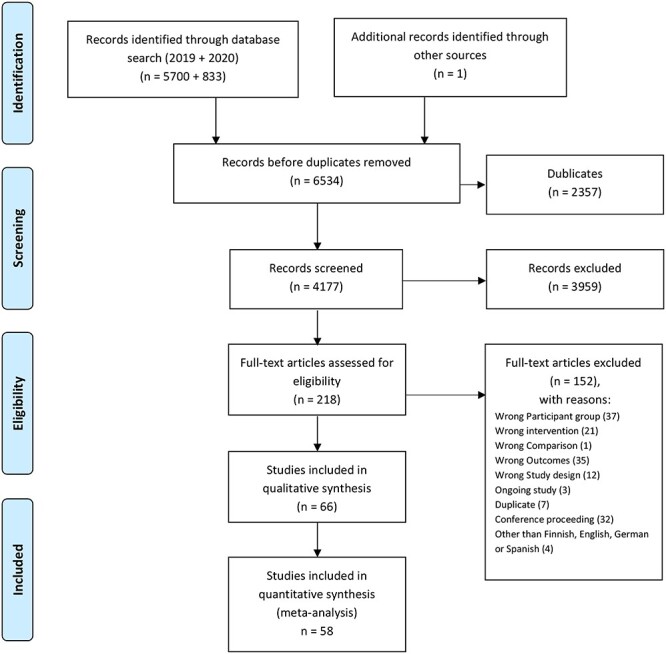
Study selection strategy flow chart.

**Figure 2:**
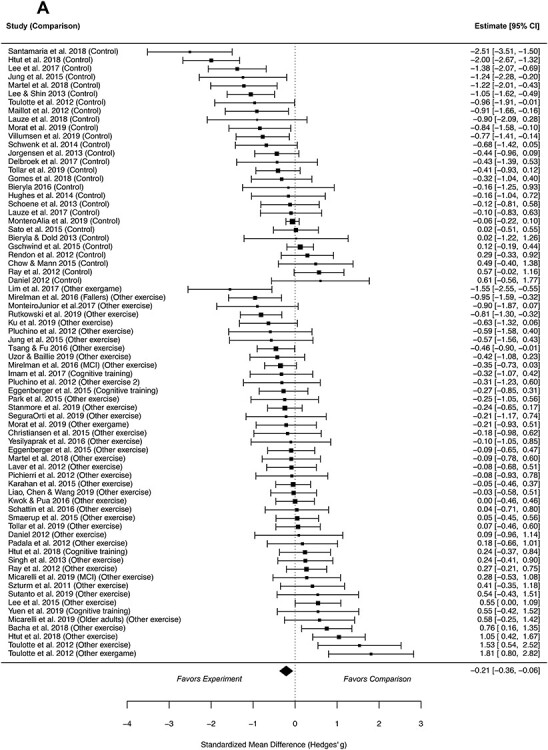

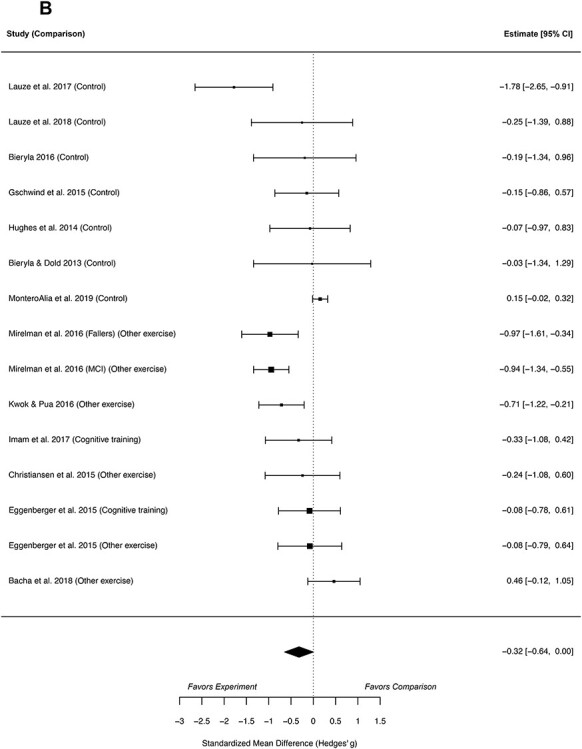
Pooled effect sizes of individual studies included in the meta-analysis: (A) After intervention (n = 58) and (B) after follow-up period (n = 13). Analysis of effects of exergaming on outcomes measuring walking compared with inactive (control) and active (other exergame, other exercise, cognitive training) comparisons by the observed effect sizes.

The search yielded 6534 studies (Fig. 1). After removal of duplicates and studies considered ineligible according to the PICOS criteria, 66 RCTs were included in the review (Fig. 1), and 58 RCTs were included in the meta-analysis (Fig. 2) and in the meta-regression (Tab. 1). Lists of the references of included and excluded studies and justifications for exclusions are included in Supplementary Material B. Detailed study characteristics of RCTs included in the qualitative synthesis are summarized in Supplementary Material C. The RCTs selected for the review were published in English, except for 1 in Spanish.^34^

#### Participants

The included studies involved 3774 participants. The range of sample sizes was from 6 to 508 (mean = 27.8, SD = 125.5) in the experimental groups and from 5 to 469 (mean = 19.2, SD = 116.0) in the comparison groups. When gender was reported, the percentage of women ranged from 0.0% to 100.0% (mean = 60.5%, SD = 25.0 in experimental group and mean = 59.2%, SD = 25.0 in comparison groups). The participants’ ages ranged from 60.5 to 87.5 years (mean = 74.3, SD = 6.8). In several studies included in the review, participant group was community-dwelling or independently living (n = 26). In other studies, participant groups were older adults with chronic disease (n = 14), mild cognitive impairment (n = 6), balance or mobility difficulties (n = 7), or participants were hospitalized or living in assistive facilities (n = 5), prefrail or frail (n = 5), sedentary (n = 2), or patients in rehabilitation or primary care centers (n = 2).

#### Interventions

Interventions were carried out at the hospital (n = 9), laboratory (n = 16), outpatient clinic (n = 11), day or welfare center or similar (n = 7), participants’ home (n = 16), or care home, assisted living facility, or similar (n = 11). The duration of interventions ranged from 2 to 26 (mean = 9, SD = 6.0) weeks with 1 to 11 (mean = 3, SD = 2) sessions per week, 11 to 90 (mean = 44, SD = 20) minutes per session. In the meta-regression, the mean value of sessions per week was used in 2 studies^35^^,^^36^ in which weekly sessions were reported as minimum sessions per week. In 12.3% of interventions, exergaming was performed in addition to other exercising. The games used in the interventions were based on technology developed to rehabilitate physical functioning (30.8%), or the games were commercially available (69.2%), such as the Nintendo Wii or the Xbox 360. In 80.0% of interventions, exergaming was supervised. Descriptions of exergame protocols and technologies used are described in Supplementary Material C. Sixteen studies had a follow-up period. Among them, 2 RCTs were excluded from the meta-analysis because walking outcomes were not remeasured after the follow-up period (Suppl. Material B). The duration of the follow-up period ranged from 3 to 36 (mean = 14, SD = 8) weeks.

#### Comparison

In 49 studies, the comparison group had other exergaming protocols or different exercising protocols, such as resistance and balance training or training with cognitive tasks (ie, active control). In 30 studies, the comparison group did not have an exercise protocol (ie, inactive control), but the group received health education, played cognitive games such as table and card games, used insoles, or continued their usual daily activities.

#### Outcomes

In the studies included in the review, walking was assessed with several tests. The results of the TUG test (post intervention n = 38, post follow-up n = 6), walking speed test (n = 9, n = 4), 2- or 6-minute walking test (n = 5, n = 1), and Functional Gait Assessment, Dynamic Gait Index, or Tinetti’s Gait test (n = 6, n = 2) were used in the studies included in the meta-analysis.

### Quality Assessment and Synthesis of Results

**Figure 3:**
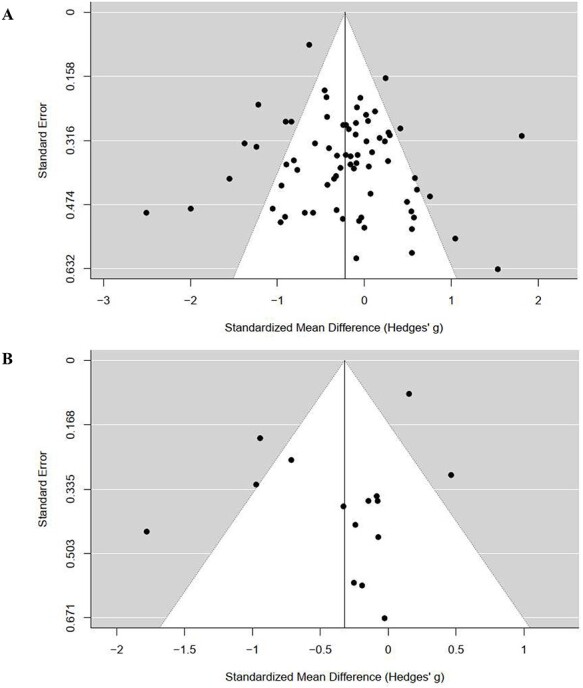
Funnel plots of studies included in the meta-analysis: (A) after intervention (n = 58) and (B) after follow-up period (n = 13).

In the studies included in the review (n = 66), the overall risk of bias was low in 2 studies, unclear in 48 studies, and high in 16 studies. High risk of bias in studies included in the quantitative synthesis had no effect on results (Tab. 1). The risk of bias in selective reporting within studies included in the meta-analysis was unclear in 55 (84.5%) studies and high in 3 (3.4%) studies. The funnel plots (Fig. 3) showed the possibility of publication bias of smaller studies favoring the experimental group. Certainty of evidence assessed with Grading of Recommendations, Assessment, Development and Evaluation was lowered because of inconsistency and imprecision within studies. The assessment is presented in the “Summary of Findings” table. The risk of bias assessments and the “Summary of Findings” table are presented in Supplementary Material C. In the multi-imputation, participants’ mean age and the logarithm of the walking speed explained 92% of the variation in the logarithm of the TUG test result in the complete cases.

**Table 1:** Results of the Meta-regression Analysis on Covariates Concerning the Study Factors (Group 1) and the High Risk of Bias Domains (Group 2)^a^

		**Model 1** ^***b***^					**Model 2** ^***c***^				
Group	Covariates	Estimated Effect Size	Lower CI	Upper CI	*P*	*R* ^2^ (%)^*d*^	Estimated Effect Size	Lower CI	Upper CI	*P*	*R* ^2^ (%)^*d*^
1	Age	0.01	−0.02	0.03	.64	0.0	0.01	−0.03	0.04	.57	14.1
	Walking performance before intervention	0.01	−0.02	0.04	.65	0.0	0.00	−0.04	0.04	.90	
	Intervention duration	0.02	−0.01	0.05	.15	0.6	0.03	−0.01	0.06	.10	
	Setting (unsupervised exergaming)	0.03	−0.35	0.41	.87	0.0	0.06	−0.33	0.45	.76	
	Sessions per week	0.05	−0.08	0.17	.45	0.0	0.07	−0.05	0.19	.26	
	Session duration	0.00	−0.01	0.01	.71	0.0	0.00	−0.02	0.01	.57	
	Type of comparison group (active/inactive)	0.48	0.20	0.77	<.001	18.6	0.50	0.19	0.81	.002	
	Technology used developed for physical rehabilitation	−0.14	−0.48	0.20	.42	0.0	−0.19	−0.53	0.16	.29	
2	Randomization process	0.27	−0.28	0.81	.34	0.0	−1.07	−1.96	−0.18	.02	27.0
	Deviations from intended interventions	0.13	−0.25	0.52	.50	0.0	−0.78	−1.45	−0.11	.02	26.0
	Missing outcome data	0.11	−0.44	0.67	.69	0.0	−1.08	−2.03	−0.14	.02	25.1
	Selection of reported results	0.16	−0.75	1.08	.73	0.0	NA	NA	NA	NA	NA
	Overall	0.15	−0.22	0.51	0.43	0.0	−0.76	−1.40	−0.11	0.02	25.8

^*a*^NA = interaction cannot be estimated because it is redundant.

^*b*^Model 1: covariates in the meta-regression model alone.

^*c*^Model 2/Group 1: covariate simultaneously with other Group 1 covariates in the meta-regression model. *R*^2^ of the model including all covariates. Model 2/Group 2: covariate simultaneously with the Group 1 covariate “type of comparison group” in the meta-regression model.

^*d*^ *R*  ^2^ (%): Negative pseudo *R*^2^ values truncated to zero.

#### After Intervention

Compared with active and inactive comparisons, exergaming had a small but significant effect on the improvement in walking (SMD = −0.21, 95% CI = −0.36 to −0.06; 3102 participants, 58 studies; moderate-quality evidence) (Fig. 2). Heterogeneity was substantial (*Q* = 245.18, *P* < .0001, *I*^2^ = 76.3%). No clear indication of asymmetry was found in the visual inspection of the funnel plot (Fig. 3) or in a statistical test for asymmetry (*P* = .48). In the meta-regression, a relationship between the exergaming effect and the type of comparison group was observed. The effect was small (SMD = 0.48, 95% CI = 0.20 to 0.77) when the type of the comparison group was evaluated alone, explaining 18.6% of the variance, and medium (SMD = 0.50, 95% CI = 0.19 to 0.81) when it was evaluated together with other covariates, explaining 14.1% of the variance (Tab. 1). Compared with the groups that had no exercise protocol (ie, inactive control), the relationship between the magnitude of the effectiveness of exergaming and covariate indicated moderate (SMD = −0.51, 95% CI = −0.74 to −0.28) benefit on walking improvements. In groups that had different exercising protocols (ie, active control), the benefit was minuscule (SMD = −0.03, 95% CI = −0.20 to 0.15).

#### After Follow-Up

Compared with active and inactive comparisons, exergaming was more or equally effective (SMD = −0.32, 95% CI = −0.64 to 0.00; 1028 participants, 13 studies; low-quality evidence) in enhancing walking (Fig. 2). Heterogeneity was substantial (*Q* = 56.94, *P* < .0001, *I*^2^ = 72.8%). Visual inspection of the funnel plot (Fig. 3) showed that the results by Lauzé et al^37^ differ from those of other studies. A statistical test did not indicate asymmetry (*P* = .95).

## Discussion

This systematic review and meta-analysis summarized the evidence from the effectiveness of exergame-based intervention on walking compared with those who had different exercising protocols or no exercise protocols among adults 60 years or older without neurological disorders. After the intervention period, the results demonstrated a small, significant effect for walking, favoring exergame interventions over comparisons. After a nonexergaming follow-up period, the results showed better effects on the exergame group. The type of comparison group (ie, inactive or active control) had the strongest association with the effect of exergaming, suggesting that compared with participants who did not exercise, participants who had exergame intervention received more benefits. Other covariates explored (participants’ age or baseline walking performance, duration or intervention setting, number of sessions per week, session duration, technology used, and high risk of bias) indicated no impact on results. Methodologic quality assessment showed mostly a moderate risk of bias within studies. The high risk of bias did not have an impact on the magnitude of effectiveness. Certainty of evidence varied from moderate to low and substantial statistical heterogeneity was observed.

Prior research among older adults had studied the effectiveness of exergame-based interventions in patients with neurological disorders^16^ by pooling outcomes measuring different characteristics of physical performance, such as muscle strength and gait^10^ or balance and gait,^17^ or with a variety of study designs.^18^ To the best of our knowledge, this is the first systematic review and meta-analysis of RCTs to focus solely on study results on outcomes measuring walking after exergame-based interventions in older adults without neurological disorders. Additionally, we also assessed the maintenance of a long-term impact from those RCTs that had a follow-up period after intervention. Only 8 of the 66 studies included in the review were excluded from the quantitative synthesis, mainly because of the availability of numerical data or because study participants were involved in 2 studies that had been included in qualitative synthesis. As a result, outcome variables measuring walking were evaluated in the meta-analysis from 58 RCTs with 3102 participants, representing a sufficient number to assess the variance between studies.^38^ Walking was comprehensively assessed from different perspectives (functional, speed, and distance), as it can be measured with several validated and standardized tests. To have a broad view of how exergaming is compared with other interventions, different comparisons (other exergame, other exercise with or without cognitive tasks, and no exercise) were included in the meta-analysis. In addition, in the meta-regression analysis, we were able to take into account different covariates, such as participants’ walking performance at the baseline, although in studies, walking was measured with different tests. Furthermore, we included comprehensive information on exergame protocols and technologies used in the studies accepted in the review. This may help researchers and rehabilitation professionals evaluate the possibilities of exergame-based interventions.

The study characteristics of the included studies show that exergaming may be used to enhance walking in a wide variety of participants and settings, as has also been noted in earlier reviews.^12^^,^^13^ Nearly one-half of the studies in our review did not focus on participants with risks or illnesses affecting their health. This may have been why the effect size remained small. A similar effect was also thought to be related to the study results of Howes et al^12^ and Vázquez et al.^10^ We are not able to fully compare our results with prior research, but there are some similarities that are good to highlight. Howes et al^12^ investigated the effect of computer gaming on functional mobility. The results of 16 studies with 670 participants favored exergaming over active or inactive control, but the effect was not significant.^12^ Comparing this result with our meta-analysis, the reason for the difference may be the large number of studies published after 2016 that we included in the analysis leading to estimates that are more extensive. Functional mobility was also assessed in the study of Donath et al,^17^ who analyzed the effect separately in active and inactive control groups. As in our results, the type of control group affected their results; when an exergaming was compared with an inactive control, the effect suggested a benefit in the exergame group, but when an exergaming was compared with an active control, the effect suggested a benefit in the control group. Nevertheless, we are not able to fully compare the results because Donath et al^17^ included Berg’s balance test in the analysis. In our study, we excluded this outcome from the analysis because we considered it a measure of balance. Vázquez et al,^10^ in turn, combined active and inactive control groups in the same manner as in our meta-analysis. They found that compared with comparison groups, the group with exergame-based interventions showed a significant positive effect on objectively measured and pooled outcomes measuring physical health. The results are consistent with the results of our study, but notably, their study combined a variety of outcomes measuring physical functioning, such as walking and muscle strength. Moreover, Vázquez et al^10^ analyzed the moderating effect of several covariates such as age, which corresponds to the covariate studied in our study. The results are conflicting; Vázquez et al^10^ found that older participants benefitted more from video game-based interventions than from comparisons. In our analysis, there was no relationship between the exergaming effect and age.

### Strengths and Limitations

The certainty and quality of this systematic review and meta-analysis is increased by the protocol registration, comprehensive search strategy, accurately defined criteria to assess search results, use of 2 reviewers in decision making in eligibility and assessments, and analysis methods used in the meta-analysis and the meta-regression. Despite these strengths, the review had some limitations that are worth noting in the generalization of the results. In many studies included in the review, sample sizes were below 20 in the experimental or comparison group, and studies had variation in interventions, type of comparison group, and walking outcomes. These factors may make assessments of the impact and effect of covariates misleading. To correct these effects, a random effect model was used in the meta-analysis, and a novel and extensive multiple imputation model was used in the meta-regression. The majority of studies (84.7%) had some concerns in selective reporting. The reason for this was mainly due to the lack of prespecified reporting and analysis methods. Future RCTs should aim to register the study, to publish a protocol article when possible, and to enhance overall methodological rigor to lower the risk of bias. Assessment of funnel plots indicated the possibility of publication bias, but it is likely that the risk remained low because of the extensive search strategy used in the literature search.

Despite these limitations, the results of this systematic review and meta-analysis provide evidence of the benefits of using exergaming to enhance walking in older adults without neurological disorders. The results are based on comprehensive research findings, more than one-half of which have been published in the previous 3 years and confirm prior research findings of the effectiveness of exergame-based training. The results represent a positive advantage in enhancing walking when a novel exercise method is used in physical rehabilitation and indicate that compared with other types of exercise interventions, similar exercise effects may be achieved with exergame-based interventions. This finding indicates that to improve walking in this age group, physiotherapists and other rehabilitation professionals may consider gamified exercises in physical rehabilitation as a promising form of exercise. Furthermore, the findings indicate that the benefits of exergame-based training are maintained in the long term.

In conclusion, older adults without neurological disorders showed improvements in walking more with exergame-based training than with active or inactive protocols. Even greater benefits were observed when exergaming groups were compared with inactive comparison groups. In addition, the benefits of exergaming are maintained in the long term. However, the favorable effect of exergame-based interventions remained small, heterogeneity between studies were substantial, and there is no clear evidence if positive effects were associated with age, baseline walking performance, and technology or specific regimen used in the exergame protocol. To strengthen the evidence, more RCTs with lower methodological variance and higher quality are needed to compare the effectiveness between gamified intervention and interventions with different exercising protocols.

## Supplementary Material

Supplementary_Material_A_pzab152

Supplementary_Material_B_pzab152

Supplementary_Material_C_pzab152

## Systematic Review Registration

This systematic review was registered prospectively in PROSPERO (CRD42020148701).
