# Supplementary material for: Effectiveness of Exergame Intervention on Walking in Older Adults: A Systematic Review and Meta-Analysis of Randomized Controlled Trials
Source: Phys Ther. 2021 Jun 21;101(9):pzab152. doi: 10.1093/ptj/pzab152 (PMC8459884; doi:10.1093/ptj/pzab152)
Supplement: Supplementary_Material_A_pzab152 [file supplementary_material_a_pzab152.pdf]

SUPLEMENTARY MATERIAL A: METHODS

In the supplementary material A are

- full electronic search strategy that was done to the MEDLINE (Ovid) database for the literature search (table A1).
- study, participant, intervention and outcome information extracted from the studies included in the review (table A2).
- obtained and confirmed data from original studies (table A3).
- conversion of given results to the mean and SD values (table A4).
- the priority list that defines the order of outcome variables measuring walking for synthesis of the results (table A5).

Table A1. The search strategy in the MEDLINE (Ovid) database.

#	Searches	Results 14.1.2019	Results 10.1.2020
1	Video Games/	4534	5104
2	Virtual Reality/	613	1426
3	Virtual Reality Exposure Therapy/	448	551
4	virtual reality.mp.	8278	9851
5	augmented reality.mp.	1396	1800
6	mixed reality.mp.	191	273
7	User-Computer Interface/	35191	36463
8	game technolog*.mp.	44	46
9	gamificati*.mp.	331	481
10	gamified.mp.	126	187
11	exergam*.mp.	496	612
12	computer gam*.mp.	1228	1323
13	wearable computing.mp.	71	84
14	digital rehabilitation.mp.	8	12
15	Wii*.mp.	949	1026
16	Sony Move*.mp.	1	1
17	Xbox*.mp.	156	176
18	X-box*.mp.	1762	1945
19	Playstation*.mp.	77	79
20	Kinect*.mp.	1006	1156
21	Intel Realsense*.mp.	3	8
22	webcam technology.mp.	3	3
23	motion detection.mp.	1262	1355
24	Motion sensor gam*.mp.	1	1
25	motion-controlled gam*.mp.	5	6
26	1 or 2 or 3 or 4 or 5 or 6 or 7 or 8 or 9 or 10 or 11 or 12 or 13 or 14 or 15 or 16 or 17 or 18 or 19 or 20 or 21 or 22 or 23 or 24 or 25	50089	54183
27	Rehabilitation/ or exp Exercise Therapy/	62230	66343
28	therapeutic exercise.mp.	910	983
29	exp Physical Therapy Modalities/	140502	148700
30	exp Exercise/	173602	187759
31	physical rehabilitation.mp.	1689	1858
32	physiotherap*.mp.	23401	25339
33	physical therap*.mp.	50567	53497
34	27 or 28 or 29 or 30 or 31 or 32 or 33	337782	360238
35	Randomized Controlled Trials as Topic/	120416	129932
36	randomized controlled trial/	474451	498448
37	Random Allocation/	97207	101853
38	Double-Blind Method/	149037	155671
39	Single Blind Method/	26115	27955
40	clinical trial/	514077	520737
41	clinical trial, phase i.pt.	18563	19850
42	clinical trial, phase ii.pt.	29941	31917
43	clinical trial, phase iii.pt.	14503	16174
44	clinical trial, phase iv.pt.	1632	1818
45	controlled clinical trial.pt.	92865	93516
46	randomized controlled trial.pt.	474451	498448
47	multicenter study.pt.	243805	264599
48	clinical trial.pt.	514077	520737
49	exp Clinical Trials as topic/	320731	335578
50	35 or 36 or 37 or 38 or 39 or 40 or 41 or 42 or 43 or 44 or 45 or 46 or 47 or 48 or 49	1271424	1338136
51	(clinical adj trial$).tw.	323540	351381
52	((singl$ or doubl$ or treb$ or tripl$) adj (blind$3 or mask$3)).tw.	161134	168770
53	PLACEBOS/	34190	34687
54	placebo$.tw.	201037	210823
55	randomly allocated.tw.	25520	27572
56	(allocated adj2 random$).tw.	28631	30811
57	51 or 52 or 53 or 54 or 55 or 56 or 57	576243	615001
58	50 or 57	1506846	1593063
59	case report.tw.	282087	300339
60	letter/	1012544	1058044
61	historical article/	349577	356143
62	59 or 60 or 61	1629568	1699138
63	58 not 62	1472735	1557049
64	26 and 34 and 63	673	765
			51

Table A2. Study, participant, intervention and outcome information extracted from the studies included in the review.

Domain	Extracted data
**Study Identification**	Country, sources of funding and author information
**Methods**	Study design and aim of the study
**Population**	Inclusion and exclusion criteria, group differences, characteristics' of participant as described in the studies
**Interventions**	Description of the interventions in experimental and comparison groups: duration, setting, type of training, used technology, guidance, exercise program (sessions in week, session time, session description, progression), adherence, follow-up procedure, additional information that review author wanted to highlight
**Outcomes**	Outcomes measuring walking measured at baseline, after intervention and when available, after follow-up period (name, type, unit of measurement, measured values, number of participants analyzed, direction (lower/upper is better), data value (endpoint/change from baseline)
	Main results of outcomes measuring walking

Table A3. Information requested from original studies (n=18) and actions made.

Study	Reason for request	Response	Actions
**Chow & Mann 2015** [1]	TUG change from baseline results reported (t test)	Endpoint Mean and SD values received via email	Endpoint values added to data extraction
**Fung et al. 2012**[2]	2MWT results reported as change% from baseline	Numerical data not received	Study excluded from meta-analysis
**Htut et al. 2018** [3]	TUG results presented graphically	Endpoint Mean and SD values received via email.	Endpoint values added to data extraction
**Khushnood et al. 2019** [4]	Participants' ages not reported	Mean age of participants under 60 years of old (information received via email)	Study excluded from the review (Reason: Wrong population)
**Lauzé et al. 2017** [5]	TUG and Walking Speed results reported as change from baseline	Endpoint Mean and SD values not received	Change values are used in meta-analysis (the groups did not differ at basline)
**Liao, Chen, & Wang 2019** [6]**; Liao, Chen, Lin, et al. 2019** [7]	Participants might be same in studies	Requested information not received	Liao et al. 2019 excluded from meta-analysis (Reason: Participant definition in trial registration)
**Lin et al. 2007** [8]	Comparison of pre- and post-intervention values for Walking Speed on four different terrains perented graphically	Numerical data not received	Study excluded from meta-analysis
**Maillot et al. 2012** [9]	TUG and 6MWT results reported as change from baseline	Endpoint Mean and SD values received via email.	Endpoint values added to data extraction
**Mirelman et al. 2016** [10]	Study participants were from three cohorts, one of which included participants with neurological disorder.	2MWT results received by three cohorts.	Two cohorts, which matched review's inclusion criteria, added to meta-analysis
**Monteiro-Junior et al. 2017** [11]	TUG and Walking speed results reported as change from baseline	Endpoint Mean and SD values received via email.	Endpoint values added to data extraction
**Ray et al. 2012** [12]	Baseline characteristics information by groups not reportes	Participant data not received	Information remains missing from narrative synthesis
**Sajid et al. 2016** [13]	6MWT results not reported	Results are not available to studies past 8 years (Information received via email)	Study excluded from meta-analysis
**Santamaría et al. 2018** [14]	TUG results reported as p-values	Endpoint Mean and SD values received via email.	Endpoint values added to data extraction
**Smaerup et al. 2015** [15]	DGI results reported as change from baseline	Endpoint Mean and SD values not received	Change values are used in meta-analysis (the groups did not differ at baseline)

**Szturm et al. 2011** [16]	TUG results reported as change from basline, Walking Speed results presented graphically	Endpoint Mean and SD values received via email	Endpoint values added to data extraction
**Tollar et al. 2019** [17]	6MWT and DGI post-intervention values not reported	Endpoint Mean and SD values received via email	Endpoint values added to data extraction
**Uzor & Baillie 2019** [18]	Walking speed results presented graphically	Endpoint Mean and SD values received via email.	Endpoint values added to data extraction

Table A4. Calculation of the mean and standard deviation (SD) values in RCTs (n=7).

Study	Reported outcomes	Values calculated	Method
**Bieryla & Dold 2013** [19]	median, lower IQR, upper IQR	mean, SD	Recommendation {q1, m, q3; n} [20]
**Bieryla 2016** [21]	median, lower IQR, upper IQR	mean, SD	Recommendation {q1, m, q3; n}[20]
**Eggenberger et al. 2015** [22]	mean, SE	SD	SD=SE*√N
**Pichierri et al. 2012** [23]	median, lower IQR, upper IQR	mean, SD	Recommendation {q1, m, q3; n}[20]
**Rendon et al. 2012** [24]	median, min, max	mean, SD	Recommendation {a, m, b; n}[20]
**Schättin et al. 2016** [25]	median, lower IQR, upper IQR	mean, SD	Recommendation {q1, m, q3; n}[20]
**Smaerup et al. 2015** [15]	mean, lower CI, upper CI	SD	SD=√N*(upper CI-lower CI)/2*tinv

Table A5. The priority list of outcome variables measuring walking in the studies included in the review.

#	Measurement	Unit of result	Direction	Incidence	Validity	Reliability	References
**1**	Timed "Up & Go" *⁾<br><br>(e.g. TUG, 8ftUG, iTUG)	second	↓	40	2-3/3	Test-retest: 4/4<br>Inter-rater: 4/4<br>Intra-rater: 4/4	[26–29]
**2**	Walking speed*⁾<br><br>(e.g. 4-m test, 10-m test, GAITRite)	milli-second, second, minute	↑	23	3/3<br>Normal pace ↑	Test-retest: 3-4/4<br>Inter-rater: 4/4	[27,29–33]
**3**	2MWT, 6MWT	feet, yard, meter	↑	16	3/3	Test-retest: 4/4<br>Inter-rater: 4/4	[29,34–36]
**4**	Functional Gait Assessment, Dynamic Gait Index, Tinetti Gait	score	↑	9	2-3/3	Test-retest: 4/4<br>Inter-rater: 3/4<br>Intra-rater: 3/4	[27,37–41]

Validity: 1/3 = small [r<.03], 2/3 = medium [r=.03-.05], 3/3 = large [r>.05] [42]
Reliability: 1/4 = poor [ICC<.05], 2/4 = moderate [ICC=.5-.75], 3/4 = good [ICC=.75-.9], 4/4 = excellent [ICC>.90] [43]
***⁾** Single-task

REFERENCES

1. Chow DHK, Mann SKF. Effect of cyber-golfing on balance amongst the elderly in Hong Kong: A pilot randomised trial. *Hong Kong J Occup Ther*. 2015;26:9-13. doi:10.1016/j.hkjot.2015.08.001

2. Fung V, Ho A, Shaffer J, Chung E, Gomez M. Use of Nintendo Wii FitTM in the rehabilitation of outpatients following total knee replacement: A preliminary randomised controlled trial. *Physiotherapy*. 2012;98:183-188. doi:10.1016/j.physio.2012.04.001

3. Htut TZC, Hiengkaew V, Jalayondeja C, Vongsirinavarat M. Effects of physical, virtual reality-based, and brain exercise on physical, cognition, and preference in older persons: A randomized controlled trial. *Eur Rev Aging Phys Act*. 2018;15:1-12. doi:10.1186/s11556-018-0199-5

4. Khushnood K, Sultan N, Mehmood R, Qureshi S, Tariq H, Amjad I. Does Wii Fit balance training improve balance and reduce fall risk in diabetic patients as compared to balance training exercises? *Rawal Med J*. 2019;44:44-48.

5. Lauzé M, Martel D, Aubertin-Leheudre M. Feasibility and effects of a physical activity program using gerontechnology in assisted living communities for Older Adults. *J Am Med Dir Assoc*. 2017;18:1069-1075. doi:10.1016/j.jamda.2017.06.030

6. Liao, Y. Y.; Chen, I. H.; Wang RY. Effects of Kinect-based exergaming on frailty status and physical performance in prefrail and frail elderly: A randomized controlled trial. *Sci Rep*. 2019;9.

7. Liao Y-Y, Chen I-H, Lin Y-J, Chen Y, Hsu W-C. Effects of virtual reality-based physical and cognitive training on executive function and dual-task gait performance in

older adults with mild cognitive impairment: A randomized control trial. *Front Aging Neurosci*. 2019;11:1-10. doi:10.3389/fnagi.2019.00162

8. Lin DH, Lin YF, Chai HM, Han YC, Jan MH. Comparison of proprioceptive functions between computerized proprioception facilitation exercise and closed kinetic chain exercise in patients with knee osteoarthritis. *Clin Rheumatol*. 2007;26:520-528. doi:10.1007/s10067-006-0324-0

9. Maillot P, Perrot A, Hartley A. Effects of interactive physical-activity video-game training on physical and cognitive function in older adults. *Psychol Aging*. 2012;27:589-600. doi:10.1037/a0026268

10. Mirelman A, Rochester L, Maidan I, et al. Addition of a non-immersive virtual reality component to treadmill training to reduce fall risk in older adults (V-TIME): A randomised controlled trial. *Lancet*. 2016;388:1170-1182. doi:10.1016/S0140-6736(16)31325-3

11. Monteiro-Junior RS, Figueiredo LFDS, Maciel-Pinheiro PDT, et al. Virtual reality–based physical exercise with exergames (PhysEx) improves mental and physical health of institutionalized older adults. *J Am Med Dir Assoc*. 2017;18:454.e1-454.e9. doi:10.1016/j.jamda.2017.01.001

12. Ray C, Melton F, Ramirez R, Keller D. The effects of a 15-week exercise intervention on fitness and postural control in older adults. *Act Adapt Aging*. 2012;36:227-241. doi:10.1080/01924788.2012.696236

13. Sajid S, Dale W, Mustian K, et al. Novel physical activity interventions for older patients with prostate cancer on hormone therapy: A pilot randomized study. *J Geriatr Oncol*. 2016;7:71-80. doi:10.1016/j.jgo.2016.02.002

14. Santamaría KG, Fonseca AS, Jiménez JM, Mora LCS. Balance, attention and concentration improvements following an exergame training program in elderly. *Retos-Nuevas Tendencias En Educ Fis Deport Y Recreacion*. 2018;2041:102-105.

15. Smaerup M, Grönvall E, Larsen SB, Laessoe U, Henriksen J-J, Damsgaard EM. Computer-assisted training as a complement in rehabilitation of patients with chronic vestibular dizziness: A randomized controlled trial. *Arch Phys Med Rehabil*. 2015;96:395-401. doi:10.1016/j.apmr.2014.10.005

16. Szturm T, Betker AL, Moussavi Z, Desai A, Goodman V. Effects of an interactive computer game exercise regimen on balance impairment in frail community-dwelling older adults: A randomized controlled trial. *Phys Ther*. 2011;91:1449-1462. doi:10.2522/ptj.20090205

17. Tollar J, Nagy F, Moizs M, Toth BE, Sanders LMJ, Hortobagyi T. Diverse exercises similarly reduce older adults' mobility limitations. *Med Sci Sports Exerc*. 2019;51:1809-1816. doi:10.1249/MSS.0000000000002001

18. Uzor S, Baillie L. Recov-R: Evaluation of a home-based tailored exergame system to reduce fall risk in seniors. *Acm Trans Comput Interact*. 2019;26. doi:10.1145/3325280

19. Bieryla KA, Dold NM. Feasibility of Wii Fit training to improve clinical measures of balance in older adults. *Clin Interv Aging*. 2013;8:775-781. doi:10.2147/CIA.S46164

20. Wan X, Wang W, Liu J, Tong T. Estimating the sample mean and standard deviation from the sample size, median, range and/or interquartile range. *BMC Med Res Methodol*. 2014;14:1-13. doi:10.1186/1471-2288-14-135

21. Bieryla KA. Xbox Kinect training to improve clinical measures of balance in older adults: A pilot study. *Aging Clin Exp Res*. 2016;28:451-457. doi:10.1007/s40520-015-

0452-y

22. Eggenberger P, Theill N, Holenstein S, Schumacher V, de Bruin ED. Multicomponent physical exercise with simultaneous cognitive training to enhance dual-task walking of older adults: A secondary analysis of a 6-month randomized controlled trial with I-year follow-up. *Clin Interv Aging*. 2015;10:1711-1732. doi:10.2147/CIA.S91997

23. Pichierri G, Murer K, de Bruin ED. A cognitive-motor intervention using a dance video game to enhance foot placement accuracy and gait under dual task conditions in older adults: A randomized controlled trial. *BMC Geriatr*. 2012;12:74. doi:10.1186/1471-2318-12-74

24. Rendon AA, Lohman EB, Thorpe D, Johnson EG, Medina E, Bradley B. The effect of virtual reality gaming on dynamic balance in older adults. *Age Ageing*. 2012;41:549-552. doi:10.1093/ageing/afs053

25. Schättin A, Arner R, Gennaro F, de Bruin ED. Adaptations of prefrontal brain activity, executive functions, and gait in healthy elderly following exergame and balance training: A randomized-controlled study. *Front Aging Neurosci*. 2016;8. doi:10.3389/fnagi.2016.00278

26. Christopher A, Kraft E, Olenick H, Kiesling R, Doty A. The reliability and validity of the Timed Up and Go as a clinical tool in individuals with and without disabilities across a lifespan: a systematic review: Psychometric properties of the Timed Up and Go. *Disabil Rehabil*. Published online 2019. doi:10.1080/09638288.2019.1682066

27. van Iersel MB, Munneke M, Esselink RAJ, Benraad CEM, Olde Rikkert MGM. Gait velocity and the Timed-Up-and-Go test were sensitive to changes in mobility in frail elderly patients. *J Clin Epidemiol*. 2008;61:186-191. doi:10.1016/j.jclinepi.2007.04.016

28. Rydwik E, Bergland A, Forsén L, Frändin K. Psychometric properties of Timed Up and Go in elderly people: A systematic review. *Phys Occup Ther Geriatr*. 2011;29:102-125. doi:10.3109/02703181.2011.564725

29. Steffen TM, Hacker TA, Mollinger L. Age- and gender-related test performance in community-dwelling elderly people:Six-minute Walk Test, Berg Balance Scale , Timed Up & Go Test, and Gait Speed. *Phys Ther*. 2002;82:128-137.

30. Bilney B, Morris M, Webster K. Concurrent related validity of the GAITRite walkway system for quantification of the spatial and temporal parameters of gait. *Gait Posture*. 2003;17:68-74. doi:10.1016/S0966-6362(02)00053-X

31. Kim H, Park I, Lee H joo, Lee O. The reliability and validity of gait speed with different walking pace and distances against general health, physical function, and chronic disease in aged adults. *J Exerc Nutr Biochem*. 2016;20:46-50. doi:10.20463/jenb.2016.09.20.3.7

32. Peters DM, Fritz SL, Krotish DE. Assessing the reliability and validity of a shorter walk test compared with the 10-Meter Walk Test for measurements of gait speed in healthy, older adults. *J Geriatr Phys Ther*. 2013;36:24-30. doi:10.1519/JPT.0b013e318248e20d

33. Rydwik E, Bergland A, Forsén L, Frändin K. Investigation into the reliability and validity of the measurement of elderly people's clinical walking speed: A systematic review. *Physiother Theory Pract*. 2012;28:238-256. doi:10.3109/09593985.2011.601804

34. Bean JF, Kiely DK, Leveille SG, et al. The 6-minute walk test in mobility-limited elders: What is being measured? *Journals Gerontol - Ser A Biol Sci Med Sci*. 2002;57. doi:10.1093/gerona/57.11.M751

35. Chan WLS, Pin TW. Reliability, validity and minimal detectable change of 2-minute

walk test, 6-minute walk test and 10-meter walk test in frail older adults with dementia. *Exp Gerontol*. 2019;115:9-18. doi:10.1016/j.exger.2018.11.001

36. Connelly DM, Thomas BK, Cliffe SJ, Perry WM, Smith RE. Clinical utility of the 2-minute walk test for older adults living in long-term care. *Physiother Canada*. 2009;61:78-87. doi:10.3138/physio.61.2.78

37. Parveen H, Noohu MM. Evaluation of psychometric properties of Tinetti performance-oriented mobility assessment scale in subjects with knee osteoarthritis. *Hong Kong Physiother J*. 2017;36:25-32. doi:10.1016/j.hkpj.2016.07.001

38. Whitney S, Wrisley D, Furman J. Concurrent validity of the Berg Balance Scale and the Dynamic Gait Index in people with vestibular dysfunction. *Physiother Res Int*. 2003;8:178-186. doi:10.1002/pri.288

39. Wrisley DM, Walker ML, Echternach JL, Strasnick B. Reliability of the Dynamic Gait Index in people with vestibular disorders. *Arch Phys Med Rehabil*. 2003;84:1528-1533. doi:10.1016/S0003-9993(03)00274-0

40. Wrisley DM, Marchetti GF, Kuharsky DK, Whitney SL. Reliability, internal consistency, and validity of data obtained with the functional gait assessment. *Phys Ther*. 2004;84:906-918. doi:10.1093/ptj/84.10.906

41. Wrisley DM, Kumar N. Functional Gait Assessment : Concurrent, discriminative, and predictive validity in community dwelling older adults. *Phys Ther*. 2010;90:1-13. doi:10.2522/ptj.20090069

42. Cohen J. A Power Primer. *Quant methods Psychol*. 1992;112:155-159. doi:10.1037/0033-2909.112.1.155

43. Koo TK, Li MY. A Guideline of Selecting and Reporting Intraclass Correlation Coefficients for Reliability Research. *J Chiropr Med*. 2016;15:155-163. doi:10.1016/j.jcm.2016.02.012
