# Supplementary material for: Effectiveness of Exergame Intervention on Walking in Older Adults: A Systematic Review and Meta-Analysis of Randomized Controlled Trials
Source: Phys Ther. 2021 Jun 21;101(9):pzab152. doi: 10.1093/ptj/pzab152 (PMC8459884; doi:10.1093/ptj/pzab152)
Supplement: Supplementary_Material_B_pzab152 [file supplementary_material_b_pzab152.pdf]

SUPLEMENTARY MATERIAL B: REFERENCES

In this supplementary material B are

- references of the studies that were included (n=66) in the review (table B1).
- references of the studies that did not met the inclusion criteria and were excluded (n=152) in the full text screening (table B2).

Table B1. Included studies (n=66) with justification for exclusion from the meta-analysis after the intervention (n=8) and after the follow-up period (n=2).

Studies included in the review	Reason for exclusion from the meta-analysis
Bacha J, Gomes G, De Freitas T, et al. Effects of kinect adventures games versus conventional physical therapy on postural control in elderly people: A randomized controlled trial. *Games Health J*. 2018;7:24-36. doi:10.1089/g4h.2017.0065	
Bieryla KA. Xbox Kinect training to improve clinical measures of balance in older adults: A pilot study. *Aging Clin Exp Res*. 2016;28:451-457. doi:10.1007/s40520-015-0452-y	
Bieryla KA, Dold NM. Feasibility of Wii Fit training to improve clinical measures of balance in older adults. *Clin Interv Aging*. 2013;8:775-781. doi:10.2147/CIA.S46164	
Chow DHK, Mann SKF. Effect of cyber-golfing on balance amongst the elderly in Hong Kong: A pilot randomised trial. *Hong Kong J Occup Ther*. 2015;26:9-13. doi:10.1016/j.hkjot.2015.08.001	
Christiansen CL, Bade MJ, Davidson BS, Dayton MR, Stevens-Lapsley JE. Effects of weight-bearing biofeedback training on functional movement patterns following total knee arthroplasty: A randomized controlled trial. *J Orthop Sports Phys Ther*. 2015;45:647-655. doi:10.2519/jospt.2015.5593	
Daniel K. Wii-hab for pre-frail older adults. *Rehabil Nurs*. 2012;37:195-201. doi:10.1002/rnj.25	
Delbroek T, Vermeylen W, Spildooren J. The effect of cognitive-motor dual task training with the biorescue force platform on cognition, balance and dual task performance in institutionalized older adults: a randomized controlled trial. *J Phys Ther Sci*. 2017;29:1137-1143. doi: 10.1589/jpts.29.1137	
Eggenberger P, Theill N, Holenstein S, Schumacher V, de Bruin ED. Multicomponent physical exercise with simultaneous cognitive training to enhance dual-task walking of older adults: A secondary analysis of a 6-month randomized controlled trial with I-year follow-up. *Clin Interv Aging*. 2015;10:1711-1732. doi:10.2147/CIA.S91997	
Fung V, Ho A, Shaffer J, Chung E, Gomez M. Use of Nintendo Wii FitTM in the rehabilitation of outpatients following total knee replacement: A preliminary randomised controlled trial. *Physiotherapy*. 2012;98:183-188. doi:10.1016/j.physio.2012.04.001	Post Intervention: Numerical data not available
Gomes GCV, Simoes M do S, Lin SM, et al. Feasibility, safety, acceptability, and functional outcomes of playing Nintendo Wii Fit PlusTM for frail older adults: A randomized feasibility clinical trial. *Maturitas*. 2018;118:20-28. doi:10.1016/j.maturitas.2018.10.002	
Gschwind YJ, Eichberg S, Ejupi A, et al. ICT-based system to predict and prevent falls (iStoppFalls): Results from an international multicenter randomized controlled trial. *Eur Rev Aging Phys Act*. 2015;12:1-11. doi:10.1186/s11556-015-0155-6	Post Follow-up: walking outcomes were not measured
Htut TZC, Hiengkaew V, Jalayondeja C, Vongsirinavarat M. Effects of physical, virtual reality-based, and brain exercise on physical, cognition, and preference in older persons: A randomized controlled trial. *Eur Rev Aging Phys Act*. 2018;15:1-12. doi:10.1186/s11556-018-0199-5	
Hughes TF, Flatt JD, Fu B, Butters MA, Chang CCH, Ganguli M. Interactive video gaming compared with health education in older adults with mild cognitive impairment: A feasibility study. *Int J Geriatr Psychiatry*. 2014;29:890-898. doi:10.1002/gps.4075	
Imam B, Miller WC, Finlayson H, Eng JJ, Jarus T. A randomized controlled trial to evaluate the feasibility of the Wii Fit for improving walking in older adults with lower limb amputation. *Clin Rehabil*. 2017;31:82-92.	

doi:10.1177/0269215515623601	
Jorgensen MG, Laessoe U, Hendriksen C, Nielsen OBF, Aagaard P. Efficacy of nintendo wii training on mechanical leg muscle function and postural balance in community-dwelling older adults: A randomized controlled trial. *Journals Gerontol - Ser A Biol Sci Med Sci*. 2013;68:845-852. doi:10.1093/gerona/gls222	
Jung D-I, Ko D-S, Jeong M-A. Kinematic effect of Nintendo Wii™ sports program exercise on obstacle gait in elderly women with falling risk. *J Phys Ther Sci*. 2015;27:1397-1400. doi:10.1589/jpts.27.1397	
Karahan AY, Tok F, Taşkın H, Küçüksaraç S, Başaran A, Yildirim P. Effects of exergames on balance, functional mobility, and quality of life of geriatrics versus home exercise programme: Randomized controlled study. *Cent Eur J Public Health*. 2015;23:S14-S18. doi:10.21101/cejph.a4081	
Ku J, Kim YJ, Cho S, Lim T, Lee HS, Kang YJ. Three-dimensional augmented reality system for balance and mobility rehabilitation in the elderly: A randomized controlled trial. *Cyberpsychology, Behav Soc Netw*. 2019;22:132-141. doi:10.1089/cyber.2018.0261	
Kwok BC, Pua YH. Effects of Wii Active exercises on fear of falling and functional outcomes in community-dwelling older adults: a randomised control trial. *Age Ageing*. 2016;45:621-628. doi:10.1093/ageing/afw108	
Lauzé M, Martel D, Agnoux A, et al. Feasibility, acceptability and effects of a home-based exercise program using a gerontechnology on physical capacities after a minor injury in community-living older adults: A pilot study. *J Nutr Heal Aging*. 2018;22:16-25. doi:10.1007/s12603-017-0938-8	
Lauzé M, Martel D, Aubertin-Leheudre M. Feasibility and effects of a physical activity program using gerontechnology in assisted living communities for Older Adults. *J Am Med Dir Assoc*. 2017;18:1069-1075. doi:10.1016/j.jamda.2017.06.030	
Laver K, George S, Ratcliffe J, et al. Use of an interactive video gaming program compared with conventional physiotherapy for hospitalised older adults: A feasibility trial. *Disabil Rehabil*. 2012;34:1802-1808. doi:10.3109/09638288.2012.662570	
Lee M, Son J, Kim J, Yoon BC. Individualized feedback-based virtual reality exercise improves older women's self-perceived health: A randomized controlled trial. *Arch Gerontol Geriatr*. 2015;61:154-160. doi:10.1016/j.archger.2015.06.010	
Lee S, Shin S. Effectiveness of virtual reality using video gaming technology in elderly adults with diabetes mellitus. *Diabetes Technol Ther*. 2013;15:489-496. doi:10.1089/dia.2013.0050	
Lee Y, Choi W, Lee K, Song C, Lee S. Virtual reality training with three-dimensional video games improves postural balance and lower extremity strength in community-dwelling older adults. *J Aging Phys Act*. 2017;25:621-627. doi:10.1123/japa.2015-0271	
Liao Y-Y, Chen I-H, Lin Y-J, Chen Y, Hsu W-C. Effects of virtual reality-based physical and cognitive training on executive function and dual-task gait performance in older adults with mild cognitive impairment: A randomized control trial. *Front Aging Neurosci*. 2019;11:1-10. doi:10.3389/fnagi.2019.00162	Post Intervention: Same participants as in Liao, Chen & Wang 2019
Liao Y-Y, Chen I-H, Wang R-Y. Effects of Kinect-based exergaming on frailty status and physical performance in prefrail and frail elderly: A randomized controlled trial. *Sci Rep*. 2019;9:1-9. doi:10.1038/s41598-019-45767-y	
Lim J, Cho JJ, Kim J, Kim Y, Yoon BC. Design of virtual reality training program for prevention of falling in the elderly: A pilot study on complex versus balance exercises. *Eur J Integr Med*. 2017;15:64-67. doi:10.1016/j.eujim.2017.09.008	

Lin DH, Lin YF, Chai HM, Han YC, Jan MH. Comparison of proprioceptive functions between computerized proprioception facilitation exercise and closed kinetic chain exercise in patients with knee osteoarthritis. *Clin Rheumatol*. 2007;26:520-528. doi:10.1007/s10067-006-0324-0	Post Intervention: Numerical data not available
Maillot P, Perrot A, Hartley A. Effects of interactive physical-activity video-game training on physical and cognitive function in older adults. *Psychol Aging*. 2012;27:589-600. doi:10.1037/a0026268	
Maillot P, Perrot A, Hartley A, Do MC. The braking force in walking: Age-related differences and improvement in older adults with exergame training. *J Aging Phys Act*. 2014;22:518-526. doi:10.1123/JAPA.2013-0001	Post intervention: Same participants as in Maillot et al. 2012
Martel D, Lauzé M, Agnoux A, et al. Comparing the effects of a home-based exercise program using a gerontechnology to a community-based group exercise program on functional capacities in older adults after a minor injury. *Exp Gerontol*. 2018;108:41-47. doi:10.1016/j.exger.2018.03.016	
Micarelli A, Viziano A, Micarelli B, Augimeri I, Alessandrini M. Vestibular rehabilitation in older adults with and without mild cognitive impairment: Effects of virtual reality using a head-mounted display. *Arch Gerontol Geriatr*. 2019;83:246-256. doi:10.1016/j.archger.2019.05.008	
Mirelman A, Rochester L, Maidan I, et al. Addition of a non-immersive virtual reality component to treadmill training to reduce fall risk in older adults (V-TIME): A randomised controlled trial. *Lancet*. 2016;388:1170-1182. doi:10.1016/S0140-6736(16)31325-3	
Monteiro-Junior RS, Figueiredo LFDS, Maciel-Pinheiro PDT, et al. Virtual reality–based physical exercise with exergames (PhysEx) improves mental and physical health of institutionalized older adults. *J Am Med Dir Assoc*. 2017;18:454.e1-454.e9. doi:10.1016/j.jamda.2017.01.001	
Montero-Alía P, Miralles-Basseda R, López-Jiménez T, et al. Controlled trial of balance training using a video game console in community-dwelling older adults. *Age Ageing*. 2019;48:506-512. doi:10.1093/ageing/afz047	
Morat M, Bakker J, Hammes V, et al. Effects of stepping exergames under stable versus unstable conditions on balance and strength in healthy community-dwelling older adults: A three-armed randomized controlled trial. *Exp Gerontol*. 2019;127:110719. doi:10.1016/j.exger.2019.110719	
Padala KP, Padala PR, Malloy TR, et al. Wii-fit for improving gait and balance in an assisted living facility: A pilot study. *J Aging Res*. 2012;2012. doi:10.1155/2012/597573	
Park EC, Kim SG, Lee CW. The effects of virtual reality game exercise on balance and gait of the elderly. *J Phys Ther Sci*. 2015;27:1157-1159. doi:10.1589/jpts.27.1157	
Pichierri G, Murer K, de Bruin ED. A cognitive-motor intervention using a dance video game to enhance foot placement accuracy and gait under dual task conditions in older adults: A randomized controlled trial. *BMC Geriatr*. 2012;12:74. doi:10.1186/1471-2318-12-74	
Pitta A, Pereira G, Lara JPR, et al. The Effects of Different Exergame Intensity Training on Walking Speed in Older Women. *Games Health J*. 2020;9:121-128. doi:10.1089/g4h.2019.0109	Post Intervention: Groups incomparable (Note. Reports outcomes from same study as Santos et al. 2019.)
Pluchino A, Lee SY, Asfour S, Roos BA, Signorile JF. Pilot study comparing changes in postural control after training using a video game balance board program and 2 standard activity-based balance intervention programs. *Arch Phys Med Rehabil*. 2012;93:1138-1146. doi:10.1016/j.apmr.2012.01.023	
Ray C, Melton F, Ramirez R, Keller D. The effects of a 15-week exercise intervention on fitness and postural control in older adults. *Act Adapt Aging*. 2012;36:227-241. doi:10.1080/01924788.2012.696236	
Rendon AA, Lohman EB, Thorpe D, Johnson EG, Medina E, Bradley B. The effect of virtual reality gaming on dynamic balance in older adults. *Age*	

Ageing. 2012;41:549-552. doi:10.1093/ageing/afs053	
Rutkowski S, Rutkowska A, Jastrzebski D, Racheniuk H, Pawelczyk W, Szczegielniak J. Effect of virtual reality-based rehabilitation on physical fitness in patients with chronic obstructive pulmonary disease. *J Hum Kinet*. 2019;69:149-157. doi:10.2478/hukin-2019-0022	
Sajid S, Dale W, Mustian K, et al. Novel physical activity interventions for older patients with prostate cancer on hormone therapy: A pilot randomized study. *J Geriatr Oncol*. 2016;7:71-80. doi:10.1016/j.jgo.2016.02.002	Post Intervention & Post follow-up: Numerical data not available
Santamaría KG, Fonseca AS, Jiménez JM, Mora LCS. Balance, attention and concentration improvements following an exergame training program in elderly. *Retos-Nuevas Tendencias En Educ Fis Deport Y Recreacion*. 2018;2041:102-105.	
Santos GOR, Wolf R, Silva MM, Rodacki ALF, Pereira G. Does exercise intensity increment in exergame promote changes in strength, functional capacity and perceptual parameters in pre-frail older women? A randomized controlled trial. *Exp Gerontol*. 2019;116:25-30. doi:10.1016/j.exger.2018.12.009	Post Intervention: Groups incomparable (Note. Reports outcomes from same study as Pitta et al. 2019)
Sato K, Kuroki K, Saiki S, Nagatomi R. Improving walking, muscle strength, and balance in the elderly with an exergame using kinect: A randomized controlled trial. *Games Health J*. 2015;4:161-167. doi:10.1089/g4h.2014.0057	
Schoene D, Lord SR, Delbaere K, Severino C, Davies TA, Smith ST. A randomized controlled pilot study of home-based step training in older people using videogame technology. *PLoS One*. 2013;8:e57734-e57734. doi:10.1371/journal.pone.0057734	
Schwenk M, Grewal GS, Honarvar B, et al. Interactive balance training integrating sensor-based visual feedback of movement performance: A pilot study in older adults. *J Neuroeng Rehabil*. 2014;11:164. doi:10.1186/1743-0003-11-164	
Schättin A, Arner R, Gennaro F, de Bruin ED. Adaptations of prefrontal brain activity, executive functions, and gait in healthy elderly following exergame and balance training: A randomized-controlled study. *Front Aging Neurosci*. 2016;8. doi:10.3389/fnagi.2016.00278	
Segura-Ortí E, Pérez-Domínguez B, Ortega-Pérez de Villar L, et al. Virtual reality exercise intradialysis to improve physical function: A feasibility randomized trial. *Scand J Med Sci Sport*. 2019;29:89-94. doi:10.1111/sms.13304	
Singh DKA, Rajaratnam BS, Palaniswamy V, Raman VP, Bong PS, Pearson H. Effects of balance-focused interactive games compared to therapeutic balance classes for older women. *Climacteric*. 2013;16:141-146. doi:10.3109/13697137.2012.664832	
Smaerup M, Grönvall E, Larsen SB, Laessoe U, Henriksen J-J, Damsgaard EM. Computer-assisted training as a complement in rehabilitation of patients with chronic vestibular dizziness: A randomized controlled trial. *Arch Phys Med Rehabil*. 2015;96:395-401. doi:10.1016/j.apmr.2014.10.005	
Smaerup M, Laessoe U, Grönvall E, Henriksen JJ, Damsgaard EM. The use of computer-assisted home exercises to preserve physical function after a vestibular rehabilitation program: A randomized controlled study. *Rehabil Res Pract*. 2016;2016. doi:10.1155/2016/7026317	Post Intervention: Same participants as in Smaerup et al. 2015 study
Stanmore EK, Mavroeidi A, de Jong LD, et al. The effectiveness and cost-effectiveness of strength and balance Exergames to reduce falls risk for people aged 55 years and older in UK assisted living facilities: A multi-centre, cluster randomised controlled trial. *BMC Med*. 2019;17:49. doi:10.1186/s12916-019-1278-9	
Sutanto YS, Makhabah DN, Aphridasari J, Doewes M, Suradi AN. Videogame assisted exercise training in patients with chronic obstructive pulmonary disease: A preliminary study. *Pulmonology*. 2019;25:275-282. doi:10.1016/j.pulmoe.2019.03.007	
Szturm T, Betker AL, Moussavi Z, Desai A, Goodman V. Effects of an interactive	

computer game exercise regimen on balance impairment in frail community-dwelling older adults: A randomized controlled trial. *Phys Ther*. 2011;91:1449-1462. doi:10.2522/ptj.20090205	
Tollar J, Nagy F, Moizs M, Toth BE, Sanders LMJ, Hortobagyi T. Diverse exercises similarly reduce older adults' mobility limitations. *Med Sci Sports Exerc*. 2019;51:1809-1816. doi:10.1249/MSS.0000000000002001	
Toulotte C, Toursel C, Olivier N. Wii Fit (R) training vs. adapted physical activities: Which one is the most appropriate to improve the balance of independent senior subjects? A randomized controlled study. *Clin Rehabil*. 2012;26:827-835. doi:10.1177/0269215511434996	
Tsang WWN, Fu ASN. Virtual reality exercise to improve balance control in older adults at risk of falling. *Hong Kong Med J*. 2016;22:S19-S22.	
Uzor S, Baillie L. Recov-R: Evaluation of a home-based tailored exergame system to reduce fall risk in seniors. *Acm Trans Comput Interact*. 2019;26. doi:10.1145/3325280	
Villumsen BR, Jorgensen MG, Frystyk J, Hørdam B, Borre M. Home-based 'exergaming' was safe and significantly improved 6-min walking distance in patients with prostate cancer: A single-blinded randomised controlled trial. *BJU Int*. 2019;124:600-608. doi:10.1111/bju.14782	Post Follow-up: walking outcomes were not measured
Yeşilyaprak SS, Yildirim MŞ, Tomruk M, Ertekin Ö, Algun ZC. Comparison of the effects of virtual reality-based balance exercises and conventional exercises on balance and fall risk in older adults living in nursing homes in Turkey. *Physiother Theory Pract*. 2016;32:191-201. doi:10.3109/09593985.2015.1138009	
Yuen HK, Lowman JD, Oster RA, de Andrade JA. Home-based pulmonary rehabilitation for patients with idiopathic pulmonary fibrosis: A pilot study. *J Cardiopulm Rehabil Prev*. 2019;39:281-284. doi:10.1097/HCR.0000000000000418	

Table B2. Excluded studies (n=152) with justification for exclusion.

Studies excluded from the review	Reason for exclusion
Alahmari KA, Sparto PJ, Marchetti GF, Redfern MS, Furman JM, Whitney SL. Comparison of virtual reality based therapy with customized vestibular physical therapy for the treatment of vestibular disorders. *Ieee Trans Neural Syst Rehabil Eng*. 2014;22:389-399.	Wrong intervention
Anderson-Hanley C, Arciero PJ, Westen SC, Nimon J, Zimmerman E. Neuropsychological benefits of stationary bike exercise and a cybercycle exergame for older adults with diabetes: An exploratory analysis. *J Diabetes Sci Technol*. 2012;6:849-857.	Wrong outcomes
Anson E, Ma L, Meetam T, et al. Trunk motion visual feedback during walking improves dynamic balance in older adults: Assessor blinded randomized controlled trial. *Gait Posture*. 2018;62:342-348.	Wrong intervention
Bade MJ. Improving strength and function after total knee arthroplasty. *Diss Abstr Int Sect B Sci Eng*. 2013;74.	Conference proceeding
Barcelos N, Shah N, Cohen K, et al. Aerobic and cognitive exercise (ACE) pilot study for older adults: Executive function improves with cognitive challenge while exergaming. *J Int Neuropsychol Soc*. 2015;21:768-779.	Wrong outcomes
Bondoc S, Hewitt P, Frey N, McQuide B, Johnson A. The effect of wii-based interventions on physical, cognitive and social functioning among pre-frail elderly persons. *Arch Phys Med Rehabil*. 2011;92:1700.	Conference proceeding
Brumels KA, Blasius T, Cortright T, Oumedian D, Solberg B. Comparison of efficacy between traditional and video game based balance programs. *Clin Kinesiol J Am Kinesiotherapy Assoc*. 2008;62:26-31.	Wrong participant group
Cacau L de AP, Oliveira GU, Maynard LG, et al. The use of the virtual reality as intervention tool in the postoperative of cardiac surgery. *Rev Bras Cir Cardiovasc*. 2013;28:281-289.	Wrong participant group
Carvalho IF de, Leme GLM, Scheicher ME. The influence of video game training with and without subpatelar bandage in mobility and gait speed on elderly female fallers. *J Aging Res*. Published online 2018:1-6.	Wrong comparator
Cawthorne D, March L, Parker D, Coolican M, Negus J. TKR-power-patient outcomes using wii enhanced rehabilitation after a total knee replacement. *Physiother (United Kingdom)*. 2015;101:eS204-eS205.	Conference proceeding
Chen C-C. Improvement in the physiological function and standing stability based on kinect multimedia for older people. *J Phys Ther Sci*. 2016;28:1343-1348.	Wrong study design
Chen P-Y, Wei S-H, Hsieh W-L, Cheen J-R, Chen L-K, Kao C-L. Lower limb power rehabilitation (LLPR) using interactive video game for improvement of balance function in older people. *Arch Gerontol Geriatr*. 2012;55:677-682.	Wrong study design
Cho GH, Hwangbo G, Shin HS. The effects of virtual reality-based balance training on balance of the elderly. J Phys Ther Sci. 2014;26:615-617.	Wrong outcomes
Chuang T-Y, Sung W-H, Lin C-Y. Application of a virtual reality-enhanced exercise protocol in patients after coronary bypass. *Arch Phys Med Rehabil*. 2005;86:1929-1932.	Wrong intervention
Collado-Mateo D, Dominguez-Munoz FJ, Adsuar JC, Merellano-Navarro E, Gusi N. Exergames for women with fibromyalgia: A randomised controlled trial to evaluate the effect on mobility skills, balance and fear of falling. *PeerJ*. 2017;5.	Wrong participant group
Correia FD, Nogueira A, Magalhaes I, et al. Home-based rehabilitation with a novel digital biofeedback system versus conventional in-person rehabilitation after total knee replacement: A feasibility study. *Sci Rep*. 2018;8.	Wrong study design
Cutter CJ, Schottenfeld RS, Moore BA, et al. A pilot trial of a videogame-based exercise program for methadone maintained patients. *J Subst Abuse Treat*. 2014;47:299-305.	Wrong participant group
da Silva Vieira AS, de Melo MCDA, Pinho ARSN., Machado JP, Mendes JGM. The effect of virtual reality on a home-based cardiac rehabilitation program on body composition, lipid profile and eating patterns: A	Wrong intervention

randomized controlled trial. *Eur J Integr Med*. 2017;9:69-78.	
Dahl-Popolizio S, Loman J, Cordes CC. Comparing outcomes of kinect videogame-based occupational/physical therapy versus usual care. *Games Health J*. 2014;3:157-161.	Wrong participant group
Daniel KM, Ray C, Cason C. Progressive functional wii-hab in pre-frail older adults. *J Am Geriatr Soc*. 2011;59:S157-S157.	Conference proceeding
Duque G, Boersma D, Loza-Diaz G, et al. Effects of balance training using a virtual-reality system in older fallers. *Clin Interv Aging*. 2013;8:257-263.	Wrong outcomes
Elshazly FAA, Nambi SG, Elnegamy TE. Comparative study on virtual reality training (VRT) over sensory motor training (SMT) in unilateral chronic osteoarthritis: A randomized control trial. *Int J Med Res Heal Sci*. 2016;5:7-16.	Wrong participant group
Fitzgerald D, Rakarnratanakul N, Smyth B, Caulfield B. Effects of a wobble board-based therapeutic exergaming system for balance training on dynamic postural stability and intrinsic motivation levels. *J Orthop Sports Phys Ther*. 2010;40:11-19.	Wrong participant group
França dos Santos F, Nunes Magalhães LHV, Nunes de Sousa FA, de Oliveira Marques C, Torres MV, Santos Leal S. Analysis of virtual reality versus functional training in fitness for elderly women. *ConScientiae Saude*. 2015;14:117-124.	Other than Finnish, English, German or Spanish
Franco JR, Jacobs K, Inzerillo C, Kluzik J. The effect of the Nintendo Wii Fit and exercise in improving balance and quality of life in community dwelling elders. *Technol Health Care*. 2012;20:95-115.	Wrong outcomes
Fu A., Gao KL, Tung AK, Tsang WW, Kwan MM. The effectiveness of exergaming training for reducing fall risk and incidence among the frail older adults with a history of falls. *Arch Phys Med Rehabil*. 2015;96:2096-2102.	Wrong outcomes
Fung V, Ho A, Shaffer J, Gomez M. The utilization of nintendo wii fit in the rehabilitation of outpatients following total knee replacements: Preliminary results of a randomized controlled trial. *Arch Phys Med Rehabil*. 2010;91:e37-e37.	Conference proceeding
Fung V, Shaffer J, Chung E, Ho A, Gomez M. The utilization of nintendo Wii FitTM in the rehabilitation of outpatients following total knee replacements: A randomized controlled trial. *Physiother (United Kingdom)*. 2011;97:eS419-eS419	Conference proceeding
Fung V, Ho A, Shaffer J, Chung E, Gomez M. Use of Nintendo Wii FitTM in the rehabilitation of outpatients following total knee replacement: A preliminary randomised controlled trial. *Physiotherapy*. 2012;98:183-188. doi:10.1016/j.physio.2012.04.001	Duplicate
Garcia AP, Gananca MM, Cusin FS, Tomaz A, Gananca FF, Caovilla HH. Vestibular rehabilitation with virtual reality in Meniere's disease. *Braz J Otorhinolaryngol*. 2013;79:366-374.	Wrong participant group
Garcia-Hernandez N, Garza-Martinez K, Parra-Vega V, Alvarez-Sanchez A, Conchas-Arteaga L. Development of an EMG-based exergaming system for isometric muscle training and its effectiveness to enhance motivation, performance and muscle strength. *Int J Hum Comput Stud*. 2019;124:44-55.	Wrong participant group
Garcia-Palacios A, Herrero R, Vizcaino Y, et al. Integrating virtual reality with activity management for the treatment of fibromyalgia: Acceptability and preliminary efficacy. *Clin J Pain*. 2015;31:564-572.	Wrong intervention
Guimaraes, A. Heart rate variability in older adults undergoing exergames and aerobic exercise training: A randomised controlled trial. *J Phys Act Health*. 2018;15:S212-S212.	Conference proceeding
Hendriks MMC, Buise MP. Interactive video games for rehabilitation in the intensive care unit: A pilot study. *J Crit Care*. 2019;51:24-25.	Wrong study design
Ho SF, Thomson A, Kerr A. Feedback integrated rehabilitation for sit-to-stand training (first): A pilot randomised controlled trial. *Age Ageing*. 2018;47:iii20-iii20	Conference proceeding
Hsia SH, Magliano LA, Sanchez H, Storer TW. "Dance dance revolution"	Conference proceeding

exergaming vs. treadmill exercise in type 2 diabetes. *Diabetes*. 2013;62:A186-A187.	
Hsieh C-C, Lin P-S, Hsu W-C, et al. The effectiveness of a virtual reality-based Tai Chi exercise on cognitive and physical function in older adults with cognitive impairment. *Dement Geriatr Cogn Disord*. 2019;46:358-370.	Duplicate
Hsieh C-C, Lin P-S, Hsu W-C, et al. The effectiveness of a virtual reality-based Tai Chi exercise on cognitive and physical function in older adults with cognitive impairment. *Dement Geriatr Cogn Disord*. Published online 2018:358-370.	Wrong study design
Hsu JK, Thibodeau R, Wong SJ, Zukiwsky D, Cecile S, Walton DM. A "Wii" bit of fun: The effects of adding Nintendo Wii Bowling to a standard exercise regimen for residents of long-term care with upper extremity dysfunction. *Physiother Theory Pract*. 2011;27:185-193.	Wrong outcomes
Hsu S-Y, Fang T-Y, Yeh S-C, Su M-C, Wang P-C, Wang VY. Three-dimensional, virtual reality vestibular rehabilitation for chronic imbalance problem caused by Meniere's disease: A pilot study. *Disabil Rehabil*. 2017;39:1601-1606.	Wrong outcomes
Ibrahim MS, Mattar AG, Elhafez SM. Efficacy of virtual reality-based balance training versus the Biodex balance system training on the body balance of adults. *J Phys Ther Sci*. 2016;28:20-26.	Wrong participant group
Jin, C.; Feng, Y. J.; Ni, Y. J.; Shan ZL. Virtual reality intervention in postoperative rehabilitation after total knee arthroplasty: A prospective and randomized controlled clinical trial. *Int J Clin Exp Med*. 2018;11:6119-6124.	Wrong intervention
Jo EA, Wu SS, Han HR, Park JJ, Park SJ, Cho KI. Effects of exergaming in postmenopausal women with high cardiovascular risk: A randomized controlled trial. *Clin Cardiol*. Published online 2019:1-8.	Wrong outcomes
Jo EA, Wu SS, Han HR, Cho KI. Impact of exergame vs. treadmill exercise on cardiorespiratory fitness, endothelial function and epicardial fat thickness in patients with high cardiovascular risk. *Eur Heart J*. 2018;39:20-20.	Conference proceeding
Karahan AY, Tok F, Yildirim P, Ordahan B, Turkoglu G, Sahin N. The effectiveness of exergames in patients with ankylosing spondylitis: A randomized controlled trial. *Adv Clin Exp Med*. 2016;25:931-936.	Wrong outcomes
Karakoc ZB, Colak TK, Sari Z, Polat MG. The effect of virtual rehabilitation added to an accelerated rehabilitation program after anterior cruciate ligament reconstruction: A randomized controlled trial. *Clin Exp Heal Sci*. 2019;9:124-129.	Wrong participant group
Karssemeijer EGA, Bossers WJR, Aaronson JA, Sanders LMJ, Kessels RPC, Rikkert M. Exergaming as a physical exercise strategy reduces frailty in people with dementia: A randomized controlled trial. *J Am Med Dir Assoc*. 2019;20:1502-1508.	Wrong intervention
Kempf K, Martin S. Autonomous use of the exercise game Wii Fit Plus improves glucometabolic control and quality of life in type 2 diabetes patients: A randomized controlled trial. *Diabetes*. 2013;62:A187-A187.	Conference proceeding
Kempf K, Martin S. Autonomous exercise game use improves metabolic control and quality of life in type 2 diabetes patients: A randomized controlled trial. *Bmc Endocr Disord*. 2013;13.	Wrong outcomes
Khalil AA, Mohamed GA, Abd El Rahman SM, Elhafez SM, Nassif, Nagui S. Effect of Wiihabilitation on strength ratio of ankle muscles in adults. *J Phys Ther Sci*. 2016;28:2862-2866.	Wrong participant group
Khushnood K, Sultan N, Mehmood R, Qureshi S, Tariq H, Amjad I. Does Wii Fit balance training improve balance and reduce fall risk in diabetic patients as compared to balance training exercises? *Rawal Med J*. 2019;44:44-48.	Wrong participant group
Kim J, Son J, Ko N, Yoon B. Unsupervised virtual reality-based exercise program improves hip muscle strength and balance control in older adults: A pilot study. *Arch Phys Med Rehabil*. 2013;94:937-943.	Wrong outcomes
Kim KJ, Heo M. Effects of virtual reality programs on balance in functional ankle instability. *J Phys Ther Sci*. 2015;27:3097-3101.	Wrong participant group

Kim KJ, Heo M. Comparison of virtual reality exercise versus conventional exercise on balance in patients with functional ankle instability: A randomized controlled trial. *J Back Musculoskelet Rehabil*. 2019;32:905-911.	Wrong participant group
Kim K, Choi B, Lim W. The efficacy of virtual reality assisted versus traditional rehabilitation intervention on individuals with functional ankle instability: A pilot randomized controlled trial. *Disabil Rehabil Assist Technol*. 2019;14:276-280.	Wrong participant group
Kim S-S, Min W-K, Kim J-H, Lee B-H. The effects of VR-based Wii Fit Yoga on physical function in middle-aged female LBP patients. *J Phys Ther Sci*. 2014;26:549-552.	Wrong intervention
Kimhy D, Vakhrusheva J, Bartels MN, et al. A single-blind randomized clinical trial of aerobic exercise in individuals with schizophrenia: Impact on brain-derived neurotrophic factor and neurocognition. *Schizophr Bull*. 2015;41:S83-S83.	Conference proceeding
Konstantinidis EI, Billis AS, Mouzakidis CA, Zilidou VI, Antoniou PE, Bamidis PD. Design, implementation, and wide pilot deployment of FitForAll: An easy to use exergaming platform improving physical fitness and life quality of senior citizens. *Ieee J Biomed Heal Informatics*. 2016;20:189-200	Wrong study design
Kotrach H, Dajczman E, Tremblay G, et al. A pilot study using virtual game system to maintain adherence to home-based exercise following pulmonary rehabilitation in chronic obstructive pulmonary disease. *Chest*. 2015;148.	Conference proceeding
Kwok BC, Pua YH. Effects of WiiActive exercises on fear of falling and functional outcomes in community-dwelling older adults: A randomised control trial. *Age Ageing*. 2016;45:621-627. doi:10.1093/ageing/afw108	Duplicate
Lee D, Sangyong L, Park J. Effects of indoor horseback riding and virtual reality exercises on the dynamic balance ability of normal healthy adults. *J Phys Ther Sci*. 2014;26:1903-1905.	Wrong study design
Lee J, Yoo H-N, Lee B-H. Effects of augmented reality-based Otago exercise on balance, gait, and physical factors in elderly women to prevent falls: A randomized controlled trial. *J Phys Ther Sci*. 2017;29:1586-1589.	Wrong intervention
Lee SW, Song CH. Virtual reality exercise improves balance of elderly persons with type 2 diabetes: A randomized controlled trial. *J Phys Ther Sci*. 2012;24:261-265.	Wrong outcomes
Leutwyler H, Hubbard E, Cooper BA, Dowling G. Impact of a pilot videogame-based physical activity program on walking speed in adults with schizophrenia. *Community Ment Health J*. 2018;54:735-739.	Wrong participant group
Liao Y-Y, Hsuan CI, Lin Y-J, Chen Y, Hsu W-C. Effects of virtual reality-based physical and cognitive training on executive function and dual-task gait performance in older adults with mild cognitive impairment: A randomized control trial. *Front Aging Neurosci*. 2019;10.	Duplicate
Makhabah D, Suradi S, Doewes M. The role of interactive game-based system in pulmonary rehabilitation of patients with COPD. *Eur Respir J*. 2015;46.	Conference proceeding
Martin-Martinez JP, Villafaina S, Collado-Mateo D, Perez-Gomez J, Gusi N. Effects of 24-week exergame intervention on physical function under single- and dual-task conditions in fibromyalgia: A randomized controlled trial. *Scand J Med Sci Sports*. 2019;29:1610-1617.	Wrong participant group
Mastel-Smith B, Duke G, He Z. A pilot randomized controlled trial examining the effects of Tai Chi and electronic tablet use on older adults' cognition and health. *J Holist Nurs*. 2019;37:163-174.	Wrong intervention
Maynard LG, de Menezes DL, Liao NS, et al. Effects of exercise training combined with virtual reality in functionality and health-related quality of life of patients on hemodialysis. *Games Health J*. 2019;8:339-348.	Wrong participant group
Mazzoleni S, Montagnani G, Vagheggini G, et al. Interactive videogame as rehabilitation tool of patients with chronic respiratory diseases: Preliminary results of a feasibility study. *Respir Med*. 2014;108:1516-1524.	Wrong outcomes

McBain T, Weston M, Crawshaw P, Haighton C, Spears I. Development of an exergame to deliver a sustained dose of high-intensity training: Formative pilot randomized trial. *Jmir Serious Games*. 2018;6:e4.	Wrong participant group
McCarthy H, Brazil ST, Greene JC, Rendell ST, Rohr LE. The impact of Wii Fit^{TM} yoga training on flexibility and heart rate. *Int Sport J*. 2013;14:67-76.	Wrong intervention
Meldrum D, Herdman S, Vance R, et al. Effectiveness of conventional versus virtual reality-based balance exercises in vestibular rehabilitation for unilateral peripheral vestibular loss: Results of a randomized controlled trial. *Arch Phys Med Rehabil*. 2015;96:1319-1328.e1.	Wrong participant group
Micarelli A, Viziano A, Augimeri I, Micarelli D, Alessandrini M. Three-dimensional head-mounted gaming task procedure maximizes effects of vestibular rehabilitation in unilateral vestibular hypofunction: A randomized controlled pilot trial. *Int J Rehabil Res*. 2017;40:325-332.	Wrong participant group
Montagnani G, Makhabah D, Vagheggini G, et al. Effectiveness of add-on interactive video games exercises in pulmonary rehabilitation programs in chronic respiratory diseases patient. *Eur Respir J*. 2013;42.	Conference proceeding
Monteiro-Junior RS, de Souza CP, Lattari E, et al. Wii-Workouts on chronic pain, physical capabilities and mood of older women: A randomized controlled double blind trial. *CNS Neurol Disord Drug Targets*. 2015;14:1157-1164.	Wrong outcomes
Morone G, Paolucci T, Luziatelli S, et al. Wii Fit is effective in women with bone loss condition associated with balance disorders: A randomized controlled trial. *Aging Clin Exp Res*. 2016;28:1187-1193.	Wrong outcomes
Morrison S, Simmons R, Colberg SR, Parson HK, Vinik AI. Supervised balance training and Wii Fit–based exercises lower falls risk in older adults with type 2 diabetes. *J Am Med Dir Assoc*. 2018;19:185.e7-185.e13.	Wrong outcomes
Mugueta-Aguinaga I, Garcia-Zapirain B. FRED: Exergame to prevent dependence and functional deterioration associated with ageing. A pilot three-week randomized controlled clinical trial. *Int J Environ Res Public Health*. 2017;14.	Wrong outcomes
Mugueta-Aguinaga I, Garcia-Zapirain B. Frailty level monitoring and analysis after a pilot six-week randomized controlled clinical trial using the FRED exergame including biofeedback supervision in an elderly day care centre. *Int J Environ Res Public Health*. 2019;16.	Wrong outcomes
Najafi B, Grewal G, Lee-Eng J, Talal TK, Menzies RA, Armstrong DG. Game-based guided exercise: Using an avatar with real-time feed back to improve postural stability in diabetic peripheral neuro pathy. *Diabetes*. 2014;63:A186-A186.	Conference proceeding
NCT01741402. Effects of training in a virtual environment in healthy elderly. *Eff Train a Virtual Environ Perform Gait, Postural Control Exec Funct Heal Elder*. Published online 2012.	Ongoing study
NCT02333214. Effectiveness of a program using video games associated with conventional physiotherapy in physical functioning in frail elderly compared to conventional physiotherapy. *Eff an Exerc Progr Using Video Games Assoc With Conv Physiother Phys Funct Frail Elder Comp to Conv Physiother randomized Clin Trial*. Published online 2015.	Ongoing study
NCT02413996. Effects of virtual reality rehabilitation in patients with total knee arthroplasty. *Eff Virtual Real Rehabil Patients With Total Knee Arthroplast a Randomised Control Trial*. Published online 2015.	Ongoing study
Nicholson VP, McKean M, Lowe J, Fawcett C, Burkett B. Six weeks of unsupervised Nintendo Wii Fit gaming is effective at improving balance in independent older adults. *J Aging Phys Act*. 2015;23:153-158.	Wrong study design
Oesch P, Kool J, Fernandez-Luque L, et al. Exergames versus self-regulated exercises with instruction leaflets to improve adherence during geriatric rehabilitation: A randomized controlled trial. *BMC Geriatr*. 2017;17.	Wrong outcomes
Ordnung M, Hoff M, Kaminski E, Villringer A, Ragert P. No overt effects of a 6-week exergame training on sensorimotor and cognitive function in older adults. A preliminary investigation. *Front Hum Neurosci*. 2017;11.	Wrong outcomes

Padala KP, Padala PR, Lensing SY, et al. Efficacy of Wii-Fit on static and dynamic balance in community dwelling older veterans: A randomized controlled pilot trial. *J Aging Res*. Published online 2017:1-9.	Wrong outcomes
Padala KP, Padala PR, Lensing SY, et al. Home-based exercise program improves balance and fear of falling in community-dwelling older adults with mild Alzheimer's disease: A pilot study. *J Alzheimers Dis*. 2017;59:565-574.	Wrong outcomes
Paolucci T, Morone G, Luziatelli S, et al. The efficacy of Wii Fit training vs. adapted physical activity in elderly subjects on balance: Preliminary results. *Ann Phys Rehabil Med*. 2014;57:e166-e166.	Conference proceeding
Parijat P, Lockhart TE, Liu J. Effects of perturbation-based slip training using a virtual reality environment on slip-induced falls. *Ann Biomed Eng*. 2015;43:958-967.	Wrong intervention
Park JH. Does cognition-specific computer training have better clinical outcomes than non-specific computer training? A single-blind, randomized controlled trial. *Clin Rehabil*. 2018;32:213-222.	Wrong outcomes
Park J, Yim J. A new approach to improve cognition, muscle strength, and postural balance in community-dwelling elderly with a 3-D virtual reality Kayak program. *Tohoku J Exp Med*. 2016;238:1-8.	Wrong intervention
Park J-H, Lee S-H, Ko D-S. The effects of the Nintendo Wii exercise program on chronic work-related low back pain in industrial workers. *J Phys Ther Sci*. 2013;25:985-988.	Wrong participant group
Park Y-J, Yoo H, Im J-H, et al. Comparison of proprioception, lower limb stability, blood pressure and ROM after proprioceptive exercise by AR exercise and therapist instruction. *Medico-Legal Updat*. 2019;19:500-506.	Wrong intervention
Parker M, Delahunty B, Heberlein N, et al. Interactive gaming consoles reduced pain during acute minor burn rehabilitation: A randomized, pilot trial. *Burns*. 2016;42:91-96.	Wrong participant group
Paukowits S, Stoggl T. Balance trainability using the Nintendo Wii balance board in sportive people. *Sportverletz Sportschaden*. 2014;28:36-43.	Wrong participant group
Pavlou M, Kanegaonkar RG, Swapp D, Bamiou DE, Slater M, Luxon LM. The effect of virtual reality on visual vertigo symptoms in patients with peripheral vestibular dysfunction: A pilot study. *J Vestib Res*. 2012;22:273-281.	Wrong intervention
Phillips JS, Fitzgerald J, Phillis D, Underwood A, Nunney I, Bath A. Vestibular rehabilitation using video gaming in adults with dizziness: A pilot study. *J Laryngol Otol*. 2018;132:202-206.	Wrong outcomes
Pichierri G, Coppe A, Lorenzetti S, Murer K, de Bruin ED. The effect of a cognitive-motor intervention on voluntary step execution under single and dual task conditions in older adults: A randomized controlled pilot study. *Clin Interv Aging*. 2012;7:175-184.	Wrong outcomes
Piqueras M, Marco E, Coll M, et al. Effectiveness of an interactive virtual telerehabilitation system in patients after total knee arthoplasty: A randomized controlled trial. *J Rehabil Med*. 2013;45:392-396.	Wrong intervention
Piqueras M, Marco E, Coll M, et al. Effectiveness of an interactive virtual telerehabilitation system in patients after total knee arthroplasty: A randomized controlled trial. *J Rehabil Med*. 2013;45:392-396.	Duplicate
Pluchino AP, Lee SY, Asfour S, Roos BA, Signorile JF. Postural control changes following training using the Wii balance program and standardized falls prevention programs. *Med Sci Sport Exerc*. 2011;43:709-709.	Conference proceeding
Pooranawatthanakul K, Foongchomcheay A. Effect of video game commercial on short term balance training in Thai elderly. *Physiother (United Kingdom)*. 2015;101:eS398-eS398.	Conference proceeding
Prasertsakul T, Kaimuk P, Chinjenpradit W, Limroongreungrat W, Charoensuk W. The effect of virtual reality-based balance training on motor learning and postural control in healthy adults: A randomized preliminary study. *Biomed Eng Online*. 2018;17.	Wrong participant group

Punt IM, Ziltener J-L, Monnin D, Allet L. Wii FitTM exercise therapy for the rehabilitation of ankle sprains: Its effect compared with physical therapy or no functional exercises at all. *Scand J Med Sci Sports*. 2016;26:816-823.	Wrong participant group
Punt IM, Armand S, Ziltener J-L, Allet L. Effect of Wii FitTM exercise therapy on gait parameters in ankle sprain patients: A randomized controlled trial. *Gait Posture*. 2017;58:52-58.	Wrong participant group
Rendon AA. Virtual reality gaming as a tool for rehabilitation in physical therapy. Published online 2011.	Wrong study design
Rezaei I, Razeghi M, Ebrahimi S, Kayedi A, Rezaeian Z. A novel virtual reality technique (Cervigame) compared to conventional proprioceptive training to treat neck pain: A randomized controlled trial. *J Biomed Phys Eng*. 2019;9:355-366.	Wrong participant group
Rodrigues E V, Gallo LH, Guimarães ATB, Melo Filho J, Luna BC, Gomes ARS. Effects of dance exergaming on depressive symptoms, fear of falling, and musculoskeletal function in fallers and nonfallers community-dwelling older women. *Rejuvenation Res*. 2018;21:518-526.	Wrong study design
Rosiak O, Szczepanik M, Woszczak M, Lucas-Grzelczyk W, Jozefowicz-Korczynska M. Effectiveness of vestibular rehabilitation in patients with vestibular dysfunction. *Med Pr*. 2019;70:545-553.	Other than Finnish, English, German or Spanish
Ruivo JMADS, Karim K, O'Shea R, et al. In-class active video game supplementation and adherence to cardiac rehabilitation. *J Cardiopulm Rehabil Prev*. 2017;37:274-278.	Wrong outcomes
Sadeghi H, Hakim MN, Hamid TA, et al. The effect of exergaming on knee proprioception in older men: a randomized controlled trial [with consumer summary]. *Arch Gerontol Geriatr 2017 Mar-Apr;69144-150*. Published online 2017.	Wrong outcomes
Sadeghi H, Hakim MN, Hamid TA, et al. The effect of exergaming on knee proprioception in older men: A randomized controlled trial. *Arch Gerontol Geriatr*. 2017;69:144-150.	Duplicate
Sajid SS, Mustian K, Dale W, et al. A novel physical activity intervention using Wii-Fit improves physical performance in older prostate cancer patients on androgen deprivation therapy: An RCT. *J Am Geriatr Soc*. 2012;60:S73-S74.	Conference proceeding
Sapi M, Domjan A, Feherne Kiss A, Pinter S. Is Kinect training superior to conventional balance training for healthy older adults to improve postural control? *Games Health J*. 2019;8:41-48.	Wrong study design
Sarig Bahat H, Croft K, Carter C, Hoddinott A, Sprecher E, Treleaven J. Remote kinematic training for patients with chronic neck pain: A randomised controlled trial. *Eur Spine J*. 2018;27:1309-1323.	Wrong participant group
Sarig Bahat H, Takasaki H, Chen X, Bet-Or Y, Treleaven J. Cervical kinematic training with and without interactive VR training for chronic neck pain: A randomized clinical trial. *Man Ther*. 2015;20:68-78.	Wrong participant group
Scanlon A-M, Meldrum D, Belton A, Magnier A, Coleman K, O'Neill D. Use of Nintendo Wii® and its effect on the balance of older adults at risk of falls: A pilot RCT. *Physiother (United Kingdom)*. 2011;97:eS1105-eS1106.	Conference proceeding
Scanlon AM, Belton A, Magnier A, Coleman K, O'Neill D, Meldrum D. Use of Nintendo Wii® and its effect on the balance of older adults at risk of falls: A pilot randomised controlled trial. *Eur Geriatr Med*. 2010;1:S156-S156.	Conference proceeding
Schega L, Hamacher D, Wagenaar RC. A comparison of effects of augmented reality and verbal information based interventions in elderly women after hip replacement. *Arch Phys Med Rehabil*. 2011;92:1734-1735.	Conference proceeding
Schumacher H, Strüwe S, Greger N, et al. Prospective, randomized trial of physical function in patients before and after haematopoietic stem cell transplantation. *Bone Marrow Transplant*. 2015;50:S219-S219.	Conference proceeding
Schumacher H, Stuwe S, Kropp P, et al. A prospective, randomized evaluation of the feasibility of exergaming on patients undergoing hematopoietic stem cell transplantation. *Bone Marrow Transplant*. 2018;53:584-590.	Wrong participant group

Schwenk M, Grewal GS, Holloway D, Muchna A, Garland L, Najafi B. Interactive sensor-based balance training in older cancer patients with chemotherapy-induced peripheral neuropathy: A randomized controlled trial. *Gerontology*. 2016;62:553-563.	Wrong participant group
Shake MC, Crandall KJ, Mathews RP, Falls DG, Dispennette AK. Efficacy of Bingocize: A game-centered mobile application to improve physical and cognitive performance in older adults. *Games Health J*. 2018;7:253-261.	Wrong intervention
Sherrington C, Hassett L, van den Berg M, et al. The effectiveness of affordable technology in rehabilitation to improve mobility and physical activity: Amount (activity and mobility using technology) rehabilitation trial. *Ann Phys Rehabil Med*. 2018;61S:e86.	Conference proceeding
Signorile JF, Pluchino A, Lee SY, Asfour SS, Roos BA. Wii fit balance produces similar improvements in balance and postural control to formalized training. *J Am Geriatr Soc*. 2011;59:S18-S18.	Conference proceeding
Sims J, Cosby N, Saliba EN, Hertel J, Saliba SA. Exergaming and static postural control in individuals with a history of lower limb injury [with consumer summary]. *J Athl Train 2013 May-Jun;48:314-325*. Published online 2013.	Wrong participant group
Singh DKA, Rajaratnam BS, Palaniswamy V, Pearson H, Raman VP, Bong PS. Participating in a virtual reality balance exercise program can reduce risk and fear of falls. *Maturitas*. 2012;73:239-243	Wrong outcomes
Soares Andrade EC, dos Melo W, de Deus Dini P, Azevedo Pinheiro H. Using Nintendo ® Wii to balance training in institutionalized older people: Pilot study. *Fisioter Bras*. 2013;14:264-267.	Other than Finnish, English, German or Spanish
Sparrer I, Thien ADD, Ilgner J, Westhofen M. Vestibular rehabilitation using the Nintendo (R) Wii Balance Board - a user-friendly alternative for central nervous compensation. *Acta Otolaryngol*. 2013;133:239-245.	Wrong participant group
Srikesavan CS, Shay B, Szturm T. Task-oriented training with computer games for people with rheumatoid arthritis or hand osteoarthritis: A feasibility randomized controlled trial. *Games Health J*. 2016;5:295-303.	Wrong participant group
Tallon G, Seilles A, Melia G, et al. Effects of the serious game Medimoov on the functional autonomy of institutionalized older adults. *Ann Phys Rehabil Med*. 2015;58:e121-e122.	Conference proceeding
Taylor L, Kerse N, Klenk J, Borotkanics R, Maddison R. Exergames to improve the mobility of long-term care residents: A cluster randomized controlled trial. *Games Health J*. 2018;7:37-42. doi:10.1089/g4h.2017.0084	Wrong intervention
Thomas JS, France CR, Applegate ME, Leitkam ST, Walkowski S. Feasibility and safety of a virtual reality dodgeball intervention for chronic low back pain: A randomized clinical trial. *J Pain*. 2016;17:1302-1317.	Wrong participant group
Tsang WWN, Fong SSM, Tung KK, Fu ASN. Is virtual reality exercise effective in reducing falls among older adults with a history of falls? *Physiother (United Kingdom)*. 2015;101:eS1539-eS1540.	Conference proceeding
Vermeylen W, Delbroek T, Spildooren J. Effects of cognitive-motor dual task training with the Bio Rescue force platform on cognition, balance and dual task performance in institutionalized older adults. *Eur Geriatr Med*. 2016;7:S156-S156.	Conference proceeding
Whyatt C, Merriman NA, Young WR, Newell FN, Craig C. A Wii bit of fun: A novel platform to deliver effective balance training to older adults. *Games Health J*. 2015;4:423-433.	Wrong outcomes
Wi SY, Kang JH, Jang JH. Clinical feasibility of exercise game for depression treatment in older women with osteoarthritis: A pilot study. *J Phys Ther Sci*. 2013;25:165-167.	Wrong outcomes
Wibelinger LM, Batista JS, Vidmar MF, Miotto C, Pasqualotti A, Schneider RH. Conventional physiotherapy vs. wiiterapia: The effects on muscle strength in elderly women with knee osteoarthritis. *ConScientiae Saude*. 2013;12:90-96.	Other than Finnish, English, German or Spanish

Wiloth S, Werner C, Lemke NC, Bauer J, Hauer K. Motor-cognitive effects of a computerized game-based training method in people with dementia: A randomized controlled trial. *Aging Ment Health*. 2018;22:1124-1135.	Wrong outcomes
Wittelsberger R, Krug S, Tittlbach S, Bos K. The influence of Nintendo-Wii(R) bowling upon residents of retirement homes. *Zeitschrift für Gerontol + Geriatr*. 2013;46:425.	Wrong outcomes
Wonjae C, Seungwon L. The effects of virtual kayak paddling exercise on postural balance, muscle performance, and cognitive function in older adults with mild cognitive impairment: A randomized controlled trial. *J Aging Phys Act*. 2019;27:861-870.	Wrong intervention
Wu Y-Z, Lin J-Y, Wu P-L, Kuo Y-F. Effects of a hybrid intervention combining exergaming and physical therapy among older adults in a long-term care facility. *Geriatr Gerontol Int*. Published online 2018.	Wrong study design
Wu Y-Z, Lin J-Y, Wu P-L, Kuo Y-F. Effects of a hybrid intervention combining exergaming and physical therapy among older adults in a long-term care facility. *Geriatr Gerontol Int*. 2019;19:147-152.	Duplicate
Yaqoob I, Khan SU. Effectiveness of balance training on quality of life in osteoporotic women. *Rawal Med J*. 2018;43:328-331.	Wrong outcomes
Yeşilyaprak SS, Şenduran M, Tomruk M, Altin Ö, Algun ZC. The effects of exercises performed with virtual reality system on balance and fall risk in the elderly. *Fiz Rehabil*. 2014;25:S73-S74.	Conference proceeding
Yilmaz Yelvar GD, Cirak Y, Dalkilinc M, Parlak Demir Y, Guner Z, Boydak A. Is physiotherapy integrated virtual walking effective on pain, function, and kinesiophobia in patients with non-specific low-back pain? Randomised controlled trial. *Eur Spine J*. 2016;26:538-545.	Wrong intervention
Yilmaz DS, Baki AE. Effect of game based exercise programs on pain, functional mobility and balance in patients with knee osteoarthritis: Randomized controlled study. *Ann Rheum Dis*. 2019;78:498-499.	Conference proceeding
Yoon JE, Lee SM, Lim HS, Kim TH, Jeon JK, Mun MH. The effects of cognitive activity combined with active extremity exercise on balance, walking activity, memory level and quality of life of an older adult sample with dementia. *J Phys Ther Sci*. 2013;25:1601-1604.	Wrong intervention
Zadro JR, Shirley D, Simic M, et al. Video-game–based exercises for older people with chronic low back pain: A randomized controlled trial (GAMEBACK). *Phys Ther*. 2019;99:14-27.	Wrong outcomes
Zhou H, Al-Ali F, Ibrahimi A, et al. Game-based non-weight bearing exercise to improve motor performance in diabetic patients undergoing hemodialysis. *Hemodial Int*. 2017;21:A53-A53.	Conference proceeding
