# Supplementary material for: Effectiveness of Exergame Intervention on Walking in Older Adults: A Systematic Review and Meta-Analysis of Randomized Controlled Trials
Source: Phys Ther. 2021 Jun 21;101(9):pzab152. doi: 10.1093/ptj/pzab152 (PMC8459884; doi:10.1093/ptj/pzab152)
Supplement: Supplementary_Material_C_pzab152 [file supplementary_material_c_pzab152.pdf]

SUPLEMENTARY MATERIAL C: RESULTS

In this supplementary material C are

- characteristics of the individual RCTs included in the review (Table C1)
- reported exergaming protocols and technologies used in the studies included in the review (Table C2).
- risk of bias in studies included in the review (Figure C1).
- summary of risk of bias across RCTs included in the meta-analysis (Figure C2).
- “summary of findings” table (Table C3).

Table C1. Characteristics of the individual RCTs included in the review.

	Participants				Interventions						
**Study and Country of Origin**	**Group**	**N**	**Age (years)**	**Female %**	**Setting(s)**	**Duration (weeks)**	**Experimental**	**Comparison(s)**	**Sessions (x/week) / Session time (min)**	**Follow-up: Duration (weeks) / Procedure**	**Outcomes measuring walking**
**Bacha et al. 2018** [1] **Brazil**	Community-dwelling	EG: 23 CG: 23	EG: 71.0 (66.0; 74.5) CG: 66.5 (65.0; 71.75)	EG: 65 CG: 83	Hospital clinic	7	Exercising with Xbox Kinect Adventures games	Conventional physical therapy exercises in a group-training program of six participants	2 / 60	4 / NR***)	FGA
**Bieryla & Dold 2013** [2] **USA**	Independently living	EG: 6 CG: 6	EG: 82.5 (1.6) CG: 80.5 (7.8)	NR	EG: Laboratory CG: Home	3	Exercising with Wii fit games	Normal daily activities	EG: 3 / 30	3 / Normal daily activities***)	TUG
**Bieryla 2016** [3] **USA**	Independently living	EG: 6 CG: 7	EG: 82 (2.4) CG: 82.6 (6.9)	NR	EG: Laboratory CG: Home	3	Exercising with Kinect-designed specific games	Normal daily activities	EG: 3 / 30	3 / Normal daily activities***)	TUG
**Chow & Mann 2015** [4] **China**	Community-dwelling	EG: 10 CG: 10	EG: 70.4 (5.4) CG: 68.0 (3.0)	EG: 70 CG: 60	Elderly day activity centre	2	Cyber-golfing training	Regular table games	7 / 30-45	NA	TUG
**Christiansen et al. 2015** [5] **USA**	TKR patients	EG: 13 CG: 13	EG: 68.2 (8.6) CG: 66.6 (8.1)	EG: 46 CG: 54	Acute care, outpatient clinic, home	6	Acute care: postoperative physical therapy protocol Home: standard of care home exercise program and Wii games	Acute care and outpatient: postoperative physical therapy protocol Home: standard of care home exercise program	Acute care: 14 Home: 7 / EG: 30 CG: NR	20 / NR***)	WS
**Daniel 2012** [6] **USA**	Pre-frail	EG: 8 CG1: 8 CG2: 7	EG: 80 (3.37) CG1: 78.13 (5.5) CG2: 72.6 (4.6)	EG: 63 CG1: 63 CG2: 57	EG, CG1: Laboratory	15	Exercising with Wii Fit games with added weight vest	CG1: Community-based seated group exercise classes CG2: Normal daily activities	EG, CG1: 3 / 45	NA	TUG, 6MWT
**Delbroek et al. 2017** [7] **Belgium**	Institutionalized with MCI	EG: 10 CG: 10	EG: 86.9 (5.6) CG: 87.5 (6.6)	EG: 80 CG: 50	Residential care centre	6	Virtual reality cognitive-motor dual-task training**)	Standard of usual care	EG: 2 / 18-30	NA	TUG, TUG Dual-task
**Eggenberger et al. 2015** [8] **Switzerland**	Independently living or community-dwelling	EG: 24 CG1: 22 CG2: 25	EG: 77.3 (6.3) CG1: 78.5 (5.1) CG2: 80.8 (4.7)	EG: 58 CG1: 73 CG2: 64	EG, CG1: Outpatient clinic + home CG:2	26	Video game dancing in groups	CG1: Treadmill walking with simultaneous verbal memory training in groups CG2: Treadmill walking in groups	2 / 60	24 / Falls calendar***)	WS (habitual, fast), WS dual-task (habitual, fast), Gait variables

					Outpatient clinic						(habitual fast), 6MWT
**Fung et al. 2012** [9] **Canada**	TKR patients	EG: 27<br>CG: 23	EG: 67.9 (9.5)<br>CG: 68.2 (12.8)	EG: 58<br>CG: 42	Outpatient department of a rehabilitation hospital	5	Wii Fit gaming after physiotherapy session	lower extremity strengthening and balance exercises after physiotherapy session	2 / 15	NA	2MWT
**Gomes et al. 2018** [10] **Brazil**	Frail	EG: 15<br>CG: 15	EG: 83 (5.87)<br>CG: 85 (6.19)	NR	EG: Outpatient clinic	7	Exercising with Wii Fit Plus games	General advice of physical activity (WHO booklet)	EG: 2 / 50	4 / NR***)	FGA
**Gschwind et al. 2015** [11] **Germany, Spain and Australia**	Community-dwelling	EG: 78<br>CG: 75	EG: 74.7 (6.7)<br>CG: 74.7 (6.0)	EG: 56<br>CG: 67	Home*)	16	Exercising with individually tailored iStoppFalls game**)	Normal daily activities	EG: 3 + 3 / 45-120	8 / Falls calendar	TUG, WS, WS dual task
**Htut et al. 2018** [12] **Thailand**	Community-dwelling	EG: 21<br>CG1: 21<br>CG2: 21<br>CG3: 21	EG: 75.8 (4.89)<br>CG1: 75.9 (5.65)<br>CG2: 75.6 (5.33)<br>CG3: 76.0 (5.22)	EG: 52<br>CG1: 38<br>CG2: 43<br>CG3: 43	Homes for the aged	8	Exercising with Xbox 360 games	CG1: Strength and balance exercising CG2: Board and card games CG3: Normal daily activities	EG, CG1, CG2: 3 / 30	NA	TUG, TUG Dual-task
**Hughes et al. 2014** [13] **USA**	MCI	EG: 10<br>CG: 10	EG: 78.5 (7.1)<br>CG: 76.2 (4.3)	EG: 80<br>CG: 60	Centrally located church	24	Exercising with Nintendo Wii games in groups	Healthy aging education in groups	1 / 90	28 / NR***)	WS
**Imam et al. 2017** [14] **Canada**	Community living individuals using the prosthesis after amputation of the lower limb	EG: 14<br>CG: 14	EG: 61.5 (50-78)<br>CG: 62.5 (50-78)	EG: 14<br>CG: 43	Outpatient clinic, home*)	4	Exercising with Nintendo Wii Fit games	Training with Wii Big Brain games	3 / 40	3 / NR***)	2MWT
**Jorgensen et al. 2013** [15] **Denmark**	Community-dwelling	EG: 28<br>CG: 30	EG: 75.9 (5.7)<br>CG: 73.7 (6.1)	EG: 68<br>CG: 70	EG: Outpatient clinic CG: Home	10	Exercising with Nintendo Wii games in pairs	Daily use of EVA shoe insole	EG: 2 /30-40	NA	TUG
**Jung et al. 2015** [16] **Republic of Korea**	At the risk for falls	EG: 8<br>CG1: 8<br>CG2: 8	EG: 74.3 (2.1)<br>CG1: 74.3 (3.5)<br>CG2: 73.6 (2.4)	100	Senior citizen centre	8	Exercising with Nintendo Wii Sport games	CG1: Lumbar stabilization exercises CG2: Normal daily activities	EG, CG1: 2 / 30	NA	TUG, Crossing velocity (CV), Maximum vertical heel clearance (MVHC)

**Karahan et al. 2015** [17] **Turkey**	Rehabilitation clinic patients	EG: 48<br>CG: 42	EG: 71.3 (6.1)<br>CG: 71.5 (4.7)	EG: 44<br>CG: 43	EG: Outpatient clinic CG: Home	6	Exercising with Xbox 360 games	Balance, stretching and strengthening exercises	5 / 30	NA	TUG
**Ku et al. 2019** [18] **Republic of Korea**	Community-dwelling, ambulatory	EG: 18<br>CG: 16	EG: 64.7 (7.27)<br>CG: 65.0 (4.77)	50	EG: Laboratory<br>CG: Home	4	Exercising with the Kinect-designed specific games**)	lower-extremity strengthening and endurance training	3 / 30	NA	TUG
**Kwok & Pua 2016** [19] **Singapore**	Moderately frail	EG: 40<br>CG: 40	EG: 70.5 (6.7)<br>CG: 69.8 (7.5)	EG: 90<br>CG: 80	Laboratory and home	12	Exercising with Nintendo Wii Active games, additional home exercises	Gym exercises in groups, additional home exercises	1 / 60	12 + 28 / Home exercises and falls calendar***)	TUG, WS, 6MWT
**Lauzé et al. 2017** [20] **Canada**	Aassisted living, moves independently within the residence	EG: 21<br>CG: 11	EG: 80.1 (7.5)<br>CG:83.2 (6.7)	EG: 71<br>CG: 91	Assisted living residence*)	12	Exercising with Jintronix gaming system**)	Normal daily activities	EG: 2 / 45	12 / NR***)	TUG, WS
**Lauzé et al. 2018** [21] **Canada**	Community living following a minor injury	EG: 6<br>CG: 6	EG: 73.17 (2.93)<br>CG: 76 (6.51)	EG: 83<br>CG: 100	Home*)	12	Exercising with Jintronix gaming system**)	Normal daily activities	EG: 2 / 50-55	12 / NR***)	TUG, WS
**Laver et al. 2012** [22] **Australia**	Hospitalised	EG: 22<br>CG: 22	EG: 85.2 (4.7)<br>CG: 84.6 (4.4)	EG: 86<br>CG: 73	Rehabilitation unit at hospital	2	Exercising with Wii Fit games	Conventional physiotherapy	5 / 25	NA	TUG
**Lee & Shin 2013** [23] **Republic of Korea**	Diabetics	EG: 27<br>CG: 28	EG: 73.78 (4.77)<br>CG: 74.29 (5.20)	EG: 74<br>CG: 68	Welfare centre	10	Exercising with PS2 games in pairs + Health education (2 times)	Health education (2 times)	EG: 2 /50	NA	TUG, WS, Gait cadence
**Lee et al. 2015** [24] **Republic of Korea**	Women	EG: 26<br>CG: 28	EG: 68.77 (4.62)<br>CG: 67.71 (4.31)	100	Laboratory	8	Exercising with Xbox 360 games	Balance, coordination and strength exercises in group	3 / 60	NA	TUG
**Lee et al. 2017** [25] **Republic of Korea**	Community-dwelling	EG: 21<br>CG: 19	EG: 76.15 (4.55)<br>CG: 75.71 (4.91)	EG: 57<br>CG: 58	Senior welfare centre	6	Exercising with Wii Fit games in pairs + fall prevention education (3 times)	Fall prevention education (3 times)	EG: 2 / 60	NA	TUG

**Liao et al.** [26] **2019 Taiwan**	MCI	EG: 18<br>CG: 16	EG: 75.5 (5.2)<br>CG: 73.1 (6.8)	EG: 61<br>CG: 75	Laboratory	12	Exercising with Kinect-designed specific games in groups**)	Combined physical and cognitive training in groups	3 / 60	NA	WS, WS Dual-task, WS Motor-task, Gait variables (Single-, Dual-, Motor-task)
**Liao, Chen & Wang** [27] **2019 Taiwan**	Pre-frail, frail	EG: 27<br>CG: 25	EG: 79.6 (8.5)<br>CG: 84.1 (5.5)	EG: 70<br>CG: 68	Laboratory	12	Exercising with Kinect-designed specific games in groups**)	Combined physical training in groups	3 / 60	NA	TUG, WS
**Lim et al. 2017** [28] **Republic of Korea**	Independent community living	EG: 10<br>CG: 10	EG: 77.30 (5.62)<br>CG: 80.8 (5.14)	EG: 60<br>CG: 70	Laboratory	5	Combined exercises with Wii Fit Plus games	Balance exercise with Wii Fit Plus games	2 / 60	NA	TUG
**Lin et al.** [29] **2007 Taiwan**	Patients with knee OA	EG: 29<br>CG1: 26<br>CG2: 26	EG: 61.6 (8.1)<br>CG1: 61.0 (7.7)<br>CG2: 62.8 (6.3)	EG: 69<br>CG1: 81<br>CG2: 81	EG, CG1: Laboratory	8	Gamified proprioception exercises**)	CG1: Closed kinect chain exercises CG2: Health education (OA)	EG,CG1: 3 / 50	NA	WS (Ground level, Stairs, Spongy Surface, Figure 8)
**Maillot et al. 2012** [30] **France**	Sedentary lifestyle	EG: 15<br>CG: 15	EG: 73.47 (4.10)<br>CG: 73.47 (3.00)	NR	EG: Laboratory	12	Exercising with Wii Fit games in pairs	Normal daily activities	EG: 2 / 60	NA	TUG, 6MWT
**Maillot et al. 2014** [31] **France**	Sedentary lifestyle	EG: 8<br>CG: 8	EG: 74.13 (4.73)<br>CG: 74.00 (2.14)	NR	EG: Outpatient	12	Exercising with Wii Fit games in pairs	Normal daily activities	EG: 2 / 60	NA	TUG, 6MWT
**Martel et al. 2018** [32] **Canada**	Community living following a minor injury	EG: 16<br>CG1: 16<br>CG2: 12	EG: 74.9 (7.1)<br>CG1: 72.9 (6.7)<br>CG2: 72.7 (6.5)	EG: 75<br>CG1: 63<br>CG2: 75	EG: Home*)<br>CG1: Community centre	12	Exercising with Jintronix gaming system**)	CG1: Combined physical training in groups CG2: Normal daily activities	EG, CG1: 2 / 55	NA	TUG, WS, SPPB walking
**Micarelli et al. 2019** [33] **Italy**	UVH / UVH + MCI	EG: 12 / 12<br>CG: 11 / 12	EG: 74.3 (4.7) / 72.5 (3.6)<br>CG: 76.9 (4.7) / 76.3 (5.5)	EG: 50 / 58<br>CG: 55 / 58	Home*) and policlinic	4	Vestibular rehabilitation and exercising with HMD games	Vestibular rehabilitation	4 / 30-45	NA	DGI
**Mirelman et al. 2016** [34] **Belgium, Israel, Italy, the Netherlands, and the UK**	Fallers / MCI	Fallers: 109<br>MCI: 43	EG: 75.4 (6.2) / 80.3 (5.2)<br>CG: 75.6 (6.2) / 74.5 (5.4)	NR	Clinical centre	6	Combined treadmill training with VR component**)	Treadmill training	3 / 45	26 / Falls calendar***)	WS during obstacle negotiation, 2MWT

**Monteiro-Junior et al. 2017** [35] **Brazil**	Living at long term care institution	EG: 9<br>CG: 9	EG: 85 (8)<br>CG: 86 (5)	67	Long term-care institution	8	Exercising with Wii Fit games	Physical exercise	2 / 30-45	NA	TUG, WS, Gait variables
**Montero-Alía et al. 2019** [36] **Spain**	Primary care centre visitors	EG:508<br>CG: 469	EG: 75.1 (72.6–78.7)<br>CG: 75.4 (72.7–78.6)	EG: 62<br>CG: 53	Nursing home	12	Exercising with Wii Fit games	Normal daily activities	EG: 2 / 30	36 / Falls calendar***)	Tinetti's gait
**Morat et al. 2019** [37] **Germany**	Community-dwelling	EG1: 15<br>CG1: 15<br>CG2: 15	EG1: 67.5 (5.1)<br>CG1: 69.7 (6.2)<br>CG2: 71.1 (5.2)	EG1: 67<br>CG1: 60<br>CG2: 60	EG1, CG1: Laboratory	8	Group exercising with Dividat Senso games under unstable conditions**)	CG1: Group exercising with Dividat Senso games under stable conditions CG2: Normal daily activities	EG1, CG1: 3 / 10-12	NA	TUG, TUG Dual-task
**Padala et al. 2012** [38] **USA**	Mild AD	EG: 11<br>CG: 11	EG: 79.3 (9.8)<br>CG: 81.6 (5.2)	73	Assisted living facility	8	Exercising with Wii Fit games	Indoor walking in groups	5 / 30	NA	TUG
**Park et al. 2015** [39] **Republic of Korea**	Community-dwelling	EG: 12<br>CG: 12	EG: 66.5 (8.1)<br>CG: 65.2 (7.9)	EG: 25<br>CG: 17	NR	8	Exercising with Wii Fit games	Ball exercises	3 / 30	NA	TUG
**Pichierri et al. 2012** [40] **Switzerland**	Residents of hostels for the aged	EG: 11<br>CG: 11	EG: 86.9 (5.1)<br>CG: 85.6 (4.2)	EG: 73<br>CG: 91	Senior citizens hostels	12	Resistance and balance training in groups and additional exercising with dancing game	Resistance and balance training in groups	2 / EG: 50-55 CG: 40	NA	WS (habitual, fast, cognitive, cognitive-fast) , Gait variables (habitual, fast, cognitive, cognitive-fast)
**Pitta et al. 2020** [41] **/ Santos et al. 2019** [42] **Brazil**	Community-dwelling, pre-frail	EG: 11<br>CG: 9	EG: 69.7 (5.6)<br>CG: 69.1 (5.0)	100	Laboratory	12	Vigorous exercising with Xbox 360 games	Moderate exercising with Xbox 360 games	3 / 40	NA	TUG, WS, WS fast, Gait variables (habitual, fast)
**Pluchino et al. 2012** [43] **USA**	Independent community living	EG: 12<br>CG1: 14<br>CG2: 14	EG: 70.72 (8.46)<br>CG1: 69.28 (6.03)<br>CG2: 76.00 (7.74)	EG: 67<br>CG1: 57<br>CG2: 64	Laboratory*)	8	Exercising with Wii Fit games	CG1: Tai Chi CG2: Standard balance exercises	2 / 60	NA	TUG, Tinetti's gait

**Ray et al. 2012** [44] **USA**	Moves independent, no falls	EG: 29 CG1: 40 CG2: 18	75	67	Community-dwelling	15	Exercising with Wii Fit games	CG1: Traditional senior fitness exercising CG2: Normal daily activities	EG, CG1: 3 / 45	NA	TUG, 6MWT
**Rendon et al. 2012** [45] **USA**	Community-dwelling, at the risk for falls	EG: 20 CG: 20	EG: 85.7 (4.3) CG: 83.3 (6.2)	NR	EG: Outpatient clinic	6	Exercising with Wii Fit games	Normal daily activities	EG: 3 / 35-45	NA	TUG
**Rutkowski et al. 2019** [46] **Poland**	Patients with COPD	EG: 34 CG: 34	EG: 60.5 (4.3) CG: 62.1 (2.9)	EG: 50 CG: 47	Hospital	2	Standard pulmonary rehabilitation in groups and additional exercising with Xbox 360 games	Standard pulmonary rehabilitation in groups	5 / EG: 75 CG: 60	NA	TUG, 6MWT
**Sajid et al. 2016** [47] **USA**	Prostate cancer patients with hormone therapy	EG: 8 CG1: 6 CG2: 5	EG: 77.5 (6.7) CG1: 75.7 (9.5) CG2: 71.8 (5)	0	Home*⁾	6	Exercising with Wii Fit games	CG1: Aerobic and resistance exercising CG2: Normal daily activities	EG, CG1: 5 / NR	6 / EG, CG1: Continued exercising***⁾	6MWT
**Santamaria et al. 2018** [48] **Costa Rica**	Senior citizens	EG: 14 CG: 13	EG: 63.21 (6.05) CG: 63.08 (5.74)	EG: 79 CG: 77	EG: Laboratory	5	Video game dancing in groups	Normal daily activities	EG: 3 / NR	NA	TUG
**Sato et al. 2015** [49] **Japan**	Locally residing	EG: 28 CG: 26	EG: 70.07 (5.35) CG: 68.5 (5.47)	EG: 79 CG: 81	EG: Laboratory	7	Exercising with Kinect-designed specific games**⁾	Normal daily activities	EG: 2-3 / 40-60	NA	WS, Gait variables
**Schoene et al. 2013** [50] **Australia**	Residents of independent-living units of a retirement village	EG: 15 CG: 17	EG: 77.5 (4.5) CG: 78.4 (4.5)	NR	EG: Home*⁾	8	Exercising with stepping game	Normal daily activities	EG: 2-3 / 15-20 (minim.)	NA	TUG, TUG dual-task
**Schwenk et al. 2014** [51] **USA**	Residents in senior living community	EG: 17 CG: 16	EG: 84.3 (7.3) CG: 84.9 (6.6)	EG: 59 CG: 69	EG: Room in senior living community	4	Exercising with specific balance games**⁾	Normal daily activities	EG: 2 / 45	NA	TUG, WS (habitual, fast), Gait variability (habitual, fast)
**Schättin et al. 2016** [52] **Switzerland**	Independently living or senior residency dwelling	EG: 13 CG: 14	EG: 80 (73 ; 83) CG: 80 (72.25 ; 81.75)	EG: 38 CG: 50	Senior residence dwelling	8	Exercising with specific Dividat games in groups**⁾	Conventional balance training in groups	3 / 30	NA	WS, Cadence and Stride length in four conditions (habitual, fast, dual- task, dual-task fast)

**Segura-Orti et al. 2019** [53] **Spain**	HD patients	EG: 9<br>CG: 9	EG: 68.3 (15.6)<br>CG: 61.8 (13.0)	EG: 33<br>CG: 44	Hospital	4	Exercising with Kinect-designed specific games	Aerobic and strengthening exercise	3 / 40	NA	WS, 6MWT
**Singh et al. 2013** [54] **Malaysia**	Community-dwelling	EG: 18<br>CG: 18	EG: 61.12 (3.72)<br>CG: 64.00 (5.88)	NR	Senior citizens´ club	6	Exercising with Wii Fit games	Balance exercises in group	2 / 40	NA	TUG
**Smaerup et al. 2015** [55] **Denmark**	Patients with vestibular dysfunction	EG:30<br>CG: 30	EG: 76.65 (7.56)<br>CG: 78.68 (6.56)	EG: 58<br>CG: 65	Hospital and home*)	16	Hospital: Rehabilitation training Home: Exercising with specific Mitii games**)	Hospital: Rehabilitation training Home: Exercising following printed instructions	Hospital: 2 Home: 7 / 20-30	NA	DGI
**Smaerup et al. 2016** [56] **Denmark**	Patients with vestibular dysfunction	EG: 28<br>CG: 29	EG: 76.39 (7.63)<br>CG: 78.93 (6.58)	EG: 57<br>CG: 63	Home*)	12	Exercising with specific Mitii games**)	Exercising following printed instructions	7 / 20-30	NA	DGI
**Stanmore et al. 2019** [57] **UK**	Dwelling in assisted living facilities	EG: 56<br>CG: 50	EG: 77.9 (8.9)<br>CG: 77.8 (10.2)	EG: 80<br>CG: 76	Sheltered housing	12	Exercising with Kinect-designed specific games**) and falls prevention exercising following program leaflet	Falls prevention exercising following program leaflet	EG: 3 / 15 (exergaming)	NA	TUG
**Sutanto et al. 2019** [58] **Indonesia**	COPD patients	EG: 10<br>CG: 10	EG: 65.1 (7.5)<br>CG: 65.6 (4.7)	EG: 10<br>CG: 0	Outpatient clinic	6	Outpatient exercising program and exercising with Wii Fit program	Outpatient exercising program	3 / EG: 60 CG: 30	NA	6MWT
**Szturm et al. 2011** [59] **Canada**	Community-dwelling individuals with balance and mobility difficulties	EG: 15<br>CG: 15	EG: 80.5 (6)<br>CG: 81 (7)	EG: 67<br>CG: 60	Geriatric day hospital	8	Exercising with specific video games**)	Strengthening and balance exercises	2 / 45	NA	TUG, WS, Gait variables
**Tollar et al. 2019** [60] **Hungary**	Mobility-limited	EG: 28<br>CG1: 27<br>CG2: 28	EG: 69.2 (2.80)<br>CG1: 70.2 (4.08)<br>CG2: 69.5 (3.67)	EG: 50<br>CG1: 56<br>CG2: 54	EG: CG1: Hospital's PT gym	5	Exercising with Xbox 360 games	CG1: Cycling CG2: Normal daily activities	EG, CG1: 5 / 60	NA	6MWT, DGI
**Toulotte et al. 2012** [61] **France**	Independently living	EG: 9<br>CG1: 9<br>CG2: 9<br>CG3: 9	EG: 72.2 (8.6)<br>CG1: 76.4 (4.7)<br>CG2: 84.2 (8.1)<br>CG3: 71.8 (8.0)	EG: 56<br>CG1: 67<br>CG2: 67<br>CG3: 56	EG, CG1, CG2: Gymnasium	20	Exercising with Wii fit games	CG1: Exercising with Wii fit games + Adapted Physical activities training CG:2 Adapted physical activities training, CG3: Normal daily activities	EG, CG1, CG2: 1 / 60	NA	Tinetti's gait

**Tsang & Fu 2016** [62] **China**	Nursing home residents with poor walking ability	EG: 39<br>CG: 40	EG: 82.3 (3.8)<br>CG: 82.0 (4.3)	EG: 59<br>CG: 63	Nursing home	6	Exercising with the Wii balance games	Conventional balance training	3 / 60	NA	TUG
**Uzor & Baillie 2019** [63] **England**	History of falls	EG: 16<br>CG: 22	EG: 76.4 (6.41)<br>CG: 75.4 (6.04)	EG: 63<br>CG: 64	Home*)	8	Exercising with tailored exergame system**) in addition to standard care	Standard care (booklet, exercise video)	3 / 30 (minim.)	NA	WS
**Villumsen et al. 2019** [64] **Denmark**	Patients with prostate cancer	EG: 23<br>CG: 23	EG: 67.6 (4.6)<br>CG: 69.8 (4.4)	0	Home*)	12	Aerobic and strength exercises with Xbox360 games	Normal daily activities	EG: 3 / 60	12 / Physical activity diary	6MWT
**Yeşilyaprak et al. 2016** [65] **Turkey**	History of falls	EG: 7<br>CG: 11	EG: 70.1 (4.0)<br>CG: 73.1 (4.5)	EG: 43<br>CG: 82	Nursing home	6	Balance training with the VR rehabilitation system**)	Conventional balance training	3 / 45-55	NA	TUG
**Yuen et al. 2019** [66] **USA**	Idiopathic Pulmonary Fibrosis patients	EG: 10<br>CG: 10	EG: 67.4 (7.4)<br>CG: 72.2 (8.4)	EG: 50<br>CG: 20	Home*)	12	Exercising with Wii fit games	Cognitive video game training	3 / 30	NA	6MWT

EG = Experimental Group, CG = Comparison Group, NR = Not reported, NA = Not Applicable

Participants: Group; MCI = Mild Cognitive Impairment, TKR = Total Knee Replacement, OA = Osteoarthritis, UVH = Unilateral Vestibular Hypofunction, AD = Alzheimer's Disease, COPD = Chronic Obstructive Pulmonary Disease, HD = Hemodialysis, Age (years); Mean (SD), Medium/Median (Q1; Q3), Median (range), Female %; Rounded to even

Interventions: HMD = Head-Mounted Displays, Setting*) = unsuperviced exergaming, Experimental, **) = Game technology used developed for physical rehabilitation, Follow-up***) = Outcomes reassessed

Outcomes measuring walking: 2MWT = Two Minute Walking Test, 6MWT = Six Minute Walking Test, FGA = Functional Gait Assessment, TUG = Timed Up & Go, WS = Walking speed

Table C2. Reported exergaming protocols and used technologies in studies (n=66) included in the review.

			Exergaming protocol				Technology	
**Study and Country of Origin**	**Type of training**	**In addition to other exercising**	**Guidance**	**Super-viced**	**Session description**	**Progression**	**Hardware**	**Games made for rehabili-tation purposes**
**Bacha et al. 2018 [1] Brazil**	Exercising with Xbox Kinect Adventures games	N	Sessions guided by a physical therapist	Y	Participants trained with four games and were allowed five attempts at each game	Players engaged in games with the goal of obtaining the highest number of adventure points that affect game progression.	360 Xbox Kinect videogame console, Kinect sensor, TV	N
**Bieryla & Dold 2013 [2] USA**	Exercising with Wii fit games	N	Session were supervised, no physical assistance	Y	Participants trained yoga (half moon, chair, warrior), aerobic (torso twists), and balance games (soccer heading, ski jump). Exercising order was: yoga, aerobic, balance x2, yoga, aerobic	Participants were challenged consistently.	Nintendo Wii, Nintendo Wii Balance Board	N
**Bieryla 2016 [3] USA**	Exercising with the Kinect-designed specific games	N	Verbal instructions for the games were provided before starting the training. No physical assistance. When necessary, participants were reminded of the goal of the game during training.	Y	Participants trained with two Kinect games (Your Shape: Fitness Evolved and Kinect adventures)	NR	Kinect for Xbox 360	N
**Chow & Mann 2015 [4] China**	Cyber-golfing training	N	Golf swing demonstration sessions (2x30 min) given by trained research assistant before experimental procedures.	Y	Participants trained with "Tiger Woods PGA Tour 13 / The 10-hole gaming mode" games and were required to finish the whole game in every session	NR	Xbox 360 Kinect	N
**Christiansen et al. 2015 [5] USA**	Weight-bearing biofeedback exercise with Wii games	N	Physical therapist gave feedback on proper performance while participant was exergaming	Y	Participants trained with Wii Fit Plus games	Depending on participant's ability level, speed was increase and dynamic, unilateral and lunging activities were added	Nintendo Wii, Nintendo Wii Balance Board	N
**Daniel 2012 [6] USA**	Exercising with Wii fit games with added weight vest	N	Sessions were directed by staff	Y	Participants wore weight vest and trained with basic games such as bowling, tennis, and boxing	Core and quadriceps muscle groups were progressively overloaded with 2% of their body weight added to the weight vest every 2 weeks	Nintendo Wii	N

**Delbroek et al. 2017** [7] **Belgium**	Virtual reality cognitive-motor dual-task training	N	Sessions were guided by a physical therapist, exergaming system gave real-time feedback on the movement of the centre of pressure	Y	While standing on platform, participants trained with nine games to train balance, weight bearing, memory, attention and dual tasking. Two 90-second break per session were allowed.	Difficulty levels were adjusted by participants' skill levels, duration of session was gradually increased from 18 to 30 minutes	BioRescue (RM Ingenierie, France; includes a platform (610 × 580 × 10 mm3)	Y
**Eggenberger et al. 2015** [8] **Switzerland**	Video game dancing in groups	Y	Sessions were guided by two trained postgraduate students	Y	Participants did aerobic endurance training with video game dancing in addition to strength and balance exercises (20 minutes each)	To achieve moderate-to-vigorous exercise intensity, treadmill speed and inclination, step frequency in DANCE, or number of sets and repetitions were adapted	Impact Dance Platforms (Positive Gaming BV, Haarlem, the Netherlands), StepMania software	N
**Fung et al. 2012** [9] **Canada**	Wii Fit gaming after physiotherapy session	Y	Sessions were guided by a physical therapist	Y	15 min Wii Fit games engaging in postural control and balance in addition to 60 min physiotherapy session	Protocol started with the 'Deep Breathing' and 'Ski Slalom' games and progressed to other games after top-level scoring	Nintendo Wii, Nintendo Wii Balance Board	N
**Gomes et al. 2018** [10] **Brazil**	Exercising with Wii Fit Plus games	N	Sessions were guided by a physical therapist	Y	Participants played block A and B games (five 2-3 min games in each block) on alternate days. Each game was played twice in each session: first attempt with the manual guidance and verbal feedback, second attempt independently	NR	Nintendo Wii	N
**Gschwind et al. 2015** [11] **Germany, Spain and Australia**	Exercising with individually tailored iStoppFalls game	N	Participants received safety guidance by an experienced researcher and they were instructed with games two times by a trained research staff: at the beginning of the training and after two weeks of training. Phone support, additional home visits, and guidance through the tablet computer were offered if required.	N	Per week, participants performed at least 3 balance gaming sessions (40 min each) and 3 muscle strength gaming sessions (15-20 min each)	Task challenges, such as narrowing base of support, adjusting speed of movement, increasing the number of repetitions, were increased	ICT-based iStoppFalls system; television, personal computer (Shuttle Barebone Slim-PC), Google TV set top box (STB) by Sony, a Microsoft Kinect (3D Depth sensor), a Senior Mobility Monitor (SMM) by Philips (3D accelerometer, barometer), Nexus 7 Android tablet	Y

**Htut et al. 2018** [12] **Thailand**	Exercising with Xbox 360 games	N	Sessions were conducted by a physical therapist	Y	In 30 min session, participants played 6 games out of ten (Light Raise, Virtual Smash, Stack'em Up, One Ball Roll, Pin Push, Super Saver, Target Kick, Play Paddle Panic, Body Bally, Bamp Bash), games involved upper and lower limb and balance training	Participants progressed to advanced levels of each game when they obtained the highest score in a previous level	Xbox 360	N
**Hughes et al. 2014** [13] **USA**	Exercising with Nintendo Wii in groups	N	Sessions were guided by interventionists	Y	After 10-15 min discussion of healthy aging topics, participants trained 60 min with "core" games (bowling, golf, tennis, baseball).	New games (e.g., Boom Blox, Wii Play, and Sports Resort) were added to the end of the session (final 15-30 min). "Wii tournaments" were held in weeks 10 and 20.	Nintendo Wii	N
**Imam et al. 2017** [14] **Canada**	Exercising with Nintendo Wii Fit games in clinic and at home	N	In clinic, a trainer conducted sessions. During home sessions, participants' were contacted once a week by a trainer to monitor safety and equipment function.	N	During sessions, participants played yoga, balance, strength training and aerobic games.	Participants progressed to advanced levels of each game when they performed well in a previous level	Nintendo Wii, Nintendo Wii Balance Board	N
**Jorgensen et al. 2013** [15] **Denmark**	Exercising with Nintendo Wii in pairs	N	Sessions were supervised by a trained physical therapist	Y	Participants played five balance games (table tilt, slalom ski, perfect 10, tight rope tension, penguin slide) for 2/3 of session's duration and muscle strengthening game (standing rowing squat) for 1/3 of the session's duration. Participants rotated between games and had 10 minute pauses.	NR	Nintendo Wii	N
**Jung et al. 2015** [16] **Republic of Korea**	Exercising with Nintendo Wii Sport games	N	Participants got various visual and audio feedback and guidance from gaming system	Y	Participants played 3 games out of 4 (Wakeboard, Frisbee dog, Jet ski, Canoe game) on a unstable floor, a 2-minute break followed every 10 minutes	NR	Nintendo Wii	N
**Karahan et al. 2015** [17] **Turkey**	Exercising with Xbox 360 Kinect Adventures, Sports and Sports Season 2 games	N	Games were instructed to participants by physicians, sessions were companied by an experienced nurse	Y	Participants played football, tennis, table tennis, skiing, golf, volleyball, and bowling games	NR	Xbox 360 Kinect, 46-inch LCD TV	N
**Ku et al. 2019** [18] **Republic of Korea**	Exercising with the Kinect-designed specific games	N	Sessions were monitored by the research assistant	Y	Participants played the balloon game and cave game for exercising hip and knee flexion/extension, and rhythm game for enhancing one-leg standing ability	The research assistant adjusted training levels individually	Microsoft Kinect sensor, 3D environment displayed on a large	Y

							screen, personal computer	
**Kwok & Pua 2016** [19] **Singapore**	Exercising with Nintendo Wii Active games, additional home balance and strengthening exercises	N	Sessions were guided and supervised by a physical therapist and a therapist assistant	Y	Participants played Wii Active games using balance board and resistance band. Exercising included cardiovascular, strengthening, calisthenics and balance training. Additionally balance and strengthening exercising was done at home on non-intervention days and during follow-up period	NR	Nintendo Wii, Nintendo Wii Balance Board	N
**Lauzé et al. 2017** [20]**, Lauzé et al. 2018** [21] **Canada**	Exercising with Jintronix gaming system	N	Mainly at the beginning of intervention, a kinesiologist supervised 6 sessions, and other time was available for individual support over the phone or in person. Participants got various visual and audio feedback and guidance from gaming system	Y/N	Participants played according to individually tailored parameters. Session included warm-up period, aerobic, resistance and balance exercises, and cool-down period.	Individual degree of difficulty was adjusted by a kinesiologist according to the Web-portal reports	A computer and a TV screen or portable computer, Jintronix software, Microsoft Kinect, a TV screen	Y
**Laver et al. 2012** [22] **Australia**	Exercising with Wii Fit games	N	Sessions were supervised by a physical therapist	Y	Participants played balance, strength and aerobic games while standing	NR	Nintendo Wii, Nintendo Wii Balance Board, wireless pointer	N
**Lee & Shin 2013** [23] **Republic of Korea**	Exercising with PS2 EyeToy games in pairs	N	Sessions were guided and helped by a research assistant (exercise trainer). Participants got various visual and audio feedback and guidance from gaming system	Y	Participants had warm-up and cool-down periods and played six 2-3 minutes games (Wishi Washi: Window Washing, Keep Ups: Heading Game, Bowling, Bubble Pop, Boot Camp, and Kung Foo) that challenged balance, strength and aerobics. Participants were allowed to take a 5-min break after 3 games.	Participants started with 4 simple games for the first 2 weeks. From the third week, more challenging games (Kung Foo, Boot Camp) were added. Kung Foo game progressed to levels that are more difficult after participant completed previous stage.	PlayStation 2, Logitech motion-tracking camera, 25-inch liquid Crystal Display monitor	N
**Lee et al. 2015** [24] **Republic of Korea**	Exercising with Xbox 360 games	N	A research assistant gave information on the exergame protocol on the first day of intervention. After that, research assistant supervised session, but did not interact with participants. Participants got various visual and audio feedback and guidance from gaming system	Y	Sessions included warm-up and cool-down exercises and games in where motions were based on tai chi. After 30 min of exercising, a 5-min break was given.	NR	Microsoft Kinect, Microsoft Xbox 360, a 1625.6mm monitor screen, Your Shape Fitness Evolved software	N

**Lee et al. 2017** [25] **Republic of Korea**	Exercising with Wii Fit games in pairs	N	Sessions were monitored and instructed by six volunteer assistant, who also encourage participants to actively exergame. An avatar providing visual and auditory feedback while exergaming.	Y	Session included warm-up and cool-down periods, and six games (jogging for gait, swordplay for agility and balance, ski jump for balance, hula-hoop for balance and lower extremity strength, tennis for balance and agility, and step dance for gait and lower extremity strength) that were played competitively as circuit training.	NR	Nintendo Wii, Nintendo Wii Balance Board, Nintendo Wii joystick, a 42-inch LCD 3D TV, polarized glasses	N
**Liao et al.** [26] **2019 Taiwan**	Exercising with Kinect-designed specific games in groups	N	Sessions were supervised by an experienced physical therapist	Y	To improve balance, stability, strength and endurance, session included Tai Chi, resistance, and aerobic exercises, and functional and cognitive tasks that simulated daily activities	Virtual tasks got more challenging after participants completed the simpler tasks.	Microsoft Kinect, VR glasses	Y
**Liao, Chen & Wang** [27] **2019 Taiwan**	Exercising with Kinect-designed specific games in groups	N	Sessions were supervised by an experienced physical therapist	Y	Session included Tai Chi, resistance and aerobic exercises from PAPAMAMA program, and balance games (window cleaning, firework hitting, goldfish grasping), 20 minutes each.	Intensity was adjusted according to heart rate (50–75% of the maximal heart rate) and perceived exertion (13 to 14, equal to “somewhat hard”)	Microsoft Kinect, Tano and LongGood software packages, a screen (230 cm × 230 cm)	Y
**Lim et al. 2017** [28] **Republic of Korea**	Combined or balance exercises with Wii Fit Plus games	N	Sessions were supervised by an experienced physical therapist, who at the first session instructed exergame protocol to participants.	Y	Session included warm-up period before exergaming that included balance, strength, flexibility, and endurance games or alternatively just balance games	NR	Nintendo Wii, Nintendo Wii Balance Board	N
**Lin et al.** [29] **2007 Taiwan**	Gamified proprioception exercises	N	Sessions were instructed by an experimenter, who provided verbal instructions and demonstrations	Y	Participants trained one leg at the time for 20 minutes, and had a 10-min break before training the other leg. Game included up-and-down and left-to-right movements that were controlled by the participant stepping onto pedals.	Games started with the slowest speed and progressed to faster speed after participants completed the slower tasks.	A personal computer, a colour computer screen, and a plantar control board (length and width: 57×57 cm, thick: 4cm, pedals: 8x9 cm)	Y
**Maillot et al. 2012** [30] **France**	Exercising with Wii Fit games in pairs	N	Sessions were supervised by a physical trainer	Y	Session was divided into three periods in where participants played sport games that included tasks that required balance, stamina, cognitive judgment and combination of variety of skills. In first period participants played Tennis or Boxing game and Bowling game in three sessions, in second period Soccer Headers, Ski Jump and Marbles games, and in final period	Participants were advised to try to increase games' level of challenge and improve their performance during the intervention	Nintendo Wii, Nintendo Wii Remote and the Nunchuk, Nintendo Wii Balance Board	N

					Ski Slalom, Hula Hoop, Trampoline and Tennis Return of Serve.			
**Maillot et al. 2014** [31] **France**	Exercising with Wii Fit games in pairs	N	NR	Y	Session was divided into two periods in where participants played sport games that included tasks that required balance and stamina. In first period, participants played Tennis or Boxing game, and in second period Soccer Headers, Ski Jump, Hula Hoop and Marbles games.	Participants were advised to try to increase games' level of challenge and improve their performance during the intervention	Nintendo Wii, Nintendo Wii Remote, Wii Nunchuk, Nintendo Wii Balance Board, a portable screen (76*102 cm)	N
**Martel et al. 2018** [32] **Canada**	Exercising with Jintronix gaming system	N	A trained kinesiologist made six in-person supervision visits (sessions 1, 2, 4, 6, 12 and 18) and follow-up calls (weeks 4 and 8).	Y/N	Session included warm-up period, aerobic, strengthening and balance exercises, and cool-down period.	Individual degree of difficulty was adjusted by a kinesiologist according to the Web-portal reports	Jintronix software, Microsoft Kinect	Y
**Micarelli et al. 2019** [33] **Italy**	Exercising with HMD games in addition to the Vestibular Rehabilitation	Y	At the beginning of protocol, participants were trained by an otoneurologist with expertise in HMD implementation.	N	Daily sessions included 20-minute exergaming while sitting on chair or sofa.	The trainers evaluated compliance, correct adjustments and performance twice a week in the clinic.	Track Speed Racing 3D game, the 5.2" display of a Windows Phone, the HMD 'Revelation' 3D VR Headset	N
**Mirelman et al. 2016** [34] **Belgium, Israel, Italy, the Netherlands, and the UK**	Combined treadmill training with VR component	N	Session were supervised by a trainer. Participants got various visual and audio feedback and guidance from gaming system	Y	During session, participants walked on treadmill in computer simulated environment that included real-life challenges, consisting of obstacles, multiple pathways, and distractors that necessitated continual adjustment of steps.	Individualized progression was performed by adjusting treadmill's speed, duration of walking bouts, and size and frequency of the virtual obstacles and the distractors.	modified Microsoft Kinect, computer, large screen	Y
**Monteiro-Junior et al. 2017** [35] **Brazil**	Exercising with Wii Fit games	N	Sessions were supervised by expert in sports medicine or physiotherapist	Y	In one session, the participant once played each of the following games: Rowing Squat, Penguim Slide, Basic Run Plus, Bump and Set, Heavy Bag, and Dance Basic 1.	NR	Nintendo Wii	N
**Montero-Alía et al. 2019** [36] **Spain**	Exercising with Wii Fit games	N	Session were guided and managed by trained personnel. Participants got various visual and audio feedback and guidance from gaming system	Y	Participants played various balance exercises while standing barefoot on the balance board.	The personnel managed the sessions so that participants did all the exercises in each session for the time specified in the protocol. The number of repetitions varied for participants according to their agility.	Nintendo Wii, Nintendo Wii Balance Board	N

**Morat et al. 2019** [37] **Germany**	Group exercising with Dividat Senso games under unstable conditions	N	A qualified study assistant guided sessions.	Y	The session included two to three pre-selected motor and cognitive games. The motor games in where the stepping was the main task were Objects, Shared, Simon, Flexi, Snake, Tetris, Habitats, Birds and Hexagon. Games with cognitive challenges were Ski and Rockect.	Progression was adjusted by increasing the degree of instability	Dividat Senso device (training platform 1.13 m*1.13 m with force sensors), screen combining Dividat Senso system with swinging Posturomed system	Y
**Padala et al. 2012** [38] **USA**	Exercising with Wii Fit games	N	Session were guided by research personnel	Y	Sessions included warm-up and cool-down exercise (walk to and from room to gaming room), and strength, yoga, and balance games, 10 minutes each.	NR	Nintendo Wii, mobile television unit	N
**Park et al. 2015** [39] **Republic of Korea**	Exercising with Wii Fit games	N	NR	Y	Sessions included training with Soccer Heading, Snowboard Slalom, and Table Tilt games, 10 minutes each.	NR	Nintendo Wii	N
**Pichierri et al. 2012** [40] **Switzerland**	Resistance and balance training in groups and additional exercising with dancing game	Y	Sessions were supervised and conducted by the investigators	Y	Session included warm-up period (5 min), physical exercises (resistance training (25 min), balance exercises (10 min)) and video game dancing (10-15 min). In dancing, four 2-3 minutes song were played with a 30 seconds break after each song.	Progression was performed thru increasing number of repetitions and the load (weight vests) (physical exercises), and beats per minute and the difficulty level (video game dancing).	TX 6000 Metal DDR Platinum Pro, modification of the StepMania	N
**Pitta et al. 2020** [41] **/ Santos et al. 2019** [42] **Brazil**	Vigorous exercising with Xbox 360 games	N	Sessions were guided by a qualified instructor guided who monitored exercise intensity and postures/movements during exergaming	Y	Session included following periods: warm-up (5 min), strength exercises (20 min), dynamic balance and cardiorespiratory exercises (10 min) and cool-down (5 min).	Progression was adjusted by increasing sets and repetitions of strength training (4 repetitions from 8 repetitions to 6 repetitions during the intervention period).	Microsoft Xbox 360, Microsoft Console, Kinect	N
**Pluchino et al. 2012** [43] **USA**	Exercising with Wii Fit games	N	Participants got various visual and audio feedback and guidance from gaming system.	N	Session included warm-up (5 min), exercising with balance games (50 min) and cool-down (5-min).	Games started with easier levels of difficulty and progressed to advanced/higher levels after participants got maximum score of the level	Nintendo Wii, Nintendo Wii Balance Board	N
**Ray et al. 2012** [44] **USA**	Exercising with Wii Fit games	N	Sessions were supervised by an assistant who supported participants ensuring their safety through gaming challenges	Y	Sessions included exercises using balance board and weighted vests.	Progression was adjusted by increasing weight of the vest every two weeks from 2 to 10 pound.	Nintendo Wii, Nintendo Wii Balance Board	N

**Rendon et al. 2012** [45] **USA**	Exercising with Wii Fit games	N	Sessions were supervised by a physical therapist who assisted participants and ensured their safety during exergaming	Y	Session included warm-up and cool-down periods, and exercising with three games that included lunges, single leg extensions and twists. Game sequence was altered week-to-week during intervention. Participants were allowed to have resting periods between games.	NR	Nintendo Wii, Nintendo Wii Remote, Nintendo Wii Balance Board	N
**Rutkowski et al. 2019** [46] **Poland**	Standard pulmonary rehabilitation in groups and additional exercising with Xbox 360 games	Y	A physiotherapist supervised sessions. Participants got instructions from gaming system.	Y	Exergaming part of the session included 15-30 minutes exercising with four Kinect Adventures games at a basic level. Gaming involved rafting, cross-country running, hitting a ball projected towards the player, and a roller-coaster ride.	The heart rate level of the exercise was measured and when the participant did not reach the heart rate specified for the exercise, the exercise was continued.	Microsoft Xbox 360, Kinect , a projector with speakers	N
**Sajid et al. 2016** [47] **USA**	Exercising with Wii Fit games	N	Participants received one teaching session given by an exercise physiologist	N	Session included individually tailored exercise program using different exercises modules of game technology	Intensity of games were increased after participant demonstrated increase in physical performance	Nintendo Wii	N
**Santamaria et al. 2018** [48] **Costa Rica**	Video game dancing in groups	N	Participants got instructions how to operate the video game.	Y	During session, participants danced 14 songs in random order at the beginner's level.	NR	Nintendo Dance Dance Revolution (DDR®), control mats in front of screen, Nintendo Wii console	N
**Sato et al. 2015** [49] **Japan**	Exercising with Kinect-designed specific games	N	A physical therapist, a student, and game development staff operated sessions. Gaming system gave audio feedback to participants.	Y	Sessions included exergaming with Apple, Tightrope, Balloon popping and One-leg standing games	The games had different levels of difficulty.	Microsoft Kinect, Microsoft Kinect SDK version 1.5, Unity version 3.4.2, a three-dimensional (3D) support tool/engine used with Kinect.	Y
**Schoene et al. 2013** [50] **Australia**	Exercising with stepping game	N	Participants was instructed and they got manual how to operate and play the stepping game. During intervention, participants were contacted four times by phone and they were able to contact research staff when they needed help.	N	Participants played stepping game, in where they synchronizing their stepping with instructions presented on the screen while listening the music they had selected. Music was not synchronized with the game.	Games started with easier levels of difficulty and progressed to advanced/higher levels after participants performed well at current level	Modified DDR Stepmania, computer, television	N

**Schwenk et al. 2014** [51] **USA**	Exercising with specific balance games	N	A study coordinator, who gave instructions of balance tasks at the first session, supervised sessions. Participants got sensor-based feedback from the gaming system.	Y	Sessions included 6 blocks with 20 cycles of exercise tasks and three series of obstacle crossing with 15 repetitions each. Participants got one-minute break between successive blocks.	By the judgement of supervisor, progression was adjusted by moving to more advanced tasks and by increasing obstacle height.	a 24-inch computer screen, game-based virtual interface (MatLab®), Psych toolbox V2.54, 5 wearable inertial sensors (LegSys™)	Y
**Schättin et al. 2016** [52] **Switzerland**	Exercising with specific Dividat games in groups	N	Session were supervised and instructed by three post graduate students.	Y	Session included warm-up and cool-down periods (5 min each), 20 minute exercising with four different games.	Progression was adjusted individually by training intensity that should achieve a moderate to vigorous training level and by increasing difficulty level of the games.	Impact Dance Platform (87.5 ×87.5× 2.5 cm), desktop computer, projector	y
**Segura-Orti et al. 2019** [53] **Spain**	Exercising with Kinect-designed specific games	N	Before first session, participants had instruction and test session. A physical therapist monitored exergaming sessions.	Y	Sessions included warm-up and cool-down sessions, 5 minutes each. 30 minutes exergaming was held in 3 minutes bouts, rest period between bouts. Intensity was held between "somewhat hard" to "hard" (RPE 13-15/20).	Progression was adjusted by increasing the number of exercise bouts (from 1 to 10) and the difficulty of game. The physical therapist adjusted game-break periods and level of difficulty depending on the participant's performance.	standard computer, a TV, Microsofts Kinect, adapted version of ACT (A la Caza del Tesoro) program	N
**Singh et al. 2013** [54] **Malaysia**	Exercising with Wii Fit games	N	NR	Y	Session included warm-up and cool-down periods, 5 min each, and 30 minutes exercising with games Ski Slalom, Table Tilt, Penguin Slide, Soccer Heading, Tight Rope Walk, Perfect 10 and Tilt City.	Games started with beginners level and progressed to advanced/expert levels after participants performed well at current level	Nintendo Wii, Nintendo Wii Balance Board	N
**Smaerup et al. 2015** [55] **Denmark**	Rehabilitation training at hospital and exercising with specific Mitii games at home	Y	Participants got oral and written instructions of Mitii home exercises. During study period, game technology instructed exercises.	Y/N	Individualized sessions included drag-and-drop and follow-the-leader exercises aiming to enhance endurance, gaze stability, reflexes, smooth-pursuit eye movements and postural control	Once a month the physical therapist contacted participants to adjust progression by increasing duration, speed, and task challenges of games	Internet-connected computer, web camera connected to a cloud-based interactive training system using the Adobe Flash technology, headband	Y
**Smaerup et al. 2016** [56] **Denmark**	Exercising with specific Mitii games at home	N	Game technology instructed exercises and registered the duration of exercising. The physical therapist followed duration and contacted	N	Daily sessions included drag-and-drop and follow-the-leader games played in standing position for 20-30 minutes.	NR	Internet-connected computer, web camera connected to a cloud-based interactive training system using the	Y

			participants if they had not trained for seven days.				Adobe Flash technology, headband	
**Stanmore et al. 2019** [57] **UK**	Exercising with Kinect-designed specific games and falls prevention exercising following program leaflet	Y	The physiotherapist advised the participants. Sessions were supervised by a physiotherapist or physiotherapist's assistant	Y	Sessions included exercising with games that suited the participant's starting level of ability and usual falls prevention exercises	Progression was tailored over the 12 weeks by increasing number of games within session, game challenge and duration.	laptop, Microsoft Kinect	Y
**Sutanto et al. 2019** [58] **Indonesia**	Outpatient exercising program and exercising with Wii Fit program	Y	Supervised session with one-to-one exergaming instruction	Y	Session included 30 min cycle exercise training at intensity of 5 on modified 10-point Borg scale and 30 min exergaming with Yoga deep breathing, Yoga half-moon, Torso twist and Free run games.	NR	Nintendo Wii, Nintendo Wii Balance Board, flatscreen TV	N
**Szturm et al. 2011** [59] **Canada**	Exercising with specific video games	N	NR	Y	Sessions included exercising with three games (Under Pressure, Memory Match and Balloon Burst) by making horizontal or vertical motions while standing on pressure mat.	Progression was adjusted individually by increasing movement amplitude, game speed, game task precision, and exercise duration.	FSA pressure mat, FSA interface box, laptop computer	Y
**Tollar et al. 2019** [60] **Hungary**	Exercising with Xbox 360 games	N	Sessions were delivered by physical therapists who were trained and supervised by the principal investigator	Y	Sessions included warm-up and cool-down, 5 min each, and exergaming with three gaming modules (Reflex Ridge, Space Pop, Just Dance), 15 min each. Participants were allowed to have 5 min rest.	Training intensity was targeted to be 80% of maximum HR. When HR deviated ±5% from target, the Polar monitor gave feedback to participant.	Xbox 360	N
**Toulotte et al. 2012** [61] **France**	Exercising with Wii Fit games	N	NR	Y	Games, such as heading soccer, ski jumping, yoga, downhill skiing, game balls and tightrope walker, were used for training, a chair in front of participant for safety.	Progression was adjusted individually with game levels	Nintendo Wii	N
**Tsang & Fu 2016** [62] **China**	Exercising with the Wii Fit balance games	N	NR	Y	The Wii Fit balance training games included Soccer Heading, Table Tilt, and Balance Bubble.	NR	Nintendo Wii, Nintendo Wii Balance Board	N
**Uzor & Baillie 2019** [63] **England**	Exercising with tailored exergame system in addition to standard care	N	Research assistant made home visit to ensure a safe environment and train participants to exergames	N	Participants trained with six exergames for strength (Pigeon Express, Horse Hurdles, Fire Rescue) and balance (River Gems, Panda Peak, Snow Flags).	Progression was adjusted by three levels of game difficulty (easy, normal, difficult).	2 IMU sensors, laptop computer, Recov-R software	Y

**Villumsen et al. 2019** [64] **Denmark**	Aerobic and strength exercises with Xbox360 Sport and Adventure games	N	A physical therapist gave individual 90 min instruction before home training exergaming	N	Sessions included warm-up and cool-down period and aerobic and strength exercises by games.	To gradually increase intensity, use of free weights (0.5, 1.0 and 2.0 kg) were added to exergaming program	Microsoft Xbox 360 Kinect	N
**Yeşilyaprak et al. 2016** [65] **Turkey**	Balance training with the VR rehabilitation system	N	Sessions were provided by a trained physical therapist. Participants followed visual and audio feedback of gaming system.	Y	Session included exergames for warm-up (5 min), training (35-45 min) and cool-down (5 min)	Progression was adjusted by closing eyes while gaming, and by reducing base of support and increasing speed, duration and challenge of games.	BTS NIRVANA VR Interactive System	Y
**Yuen et al. 2019** [66] **USA**	Exercising with Wii Fit games	N	To support of participants, research assistant contacted them week after baseline assessment and once a month during intervention	N	Session included exergaming with intensity of moderate to heavy (3 to 5 on 10-point Borg scale)	NR	Nintendo Wii U, Nintendo Balance board	N

Y = Yes, N = No, NR = Not reported

Figure C1. Risk of bias in studies (n=66) included in the review: (A) Randomization process, (B) Deviations from the intended interventions, (C) Missing outcome data, (D) Measurement of the outcome, (E) Selection of the reported results, (F) Overall.

	Risk of Bias					
Study or Subgroup	A	B	C	D	E	F
Bacha et al. 2018	+	+	+	+	?	?
Bieryla & Dold 2013	?	−	+	?	?	−
Bieryla 2016	?	?	+	?	?	?
Chow & Mann2015	?	?	+	?	?	?
Christiansen et al. 2015	+	+	+	+	?	?
Daniel 2012	−	−	+	?	?	−
Delbroek et al. 2017	?	?	+	+	?	?
Eggenberger et al. 2015	?	+	+	?	?	?
Fung et al. 2012	+	+	+	+	+	+
Gomes et al. 2018	+	+	+	+	?	?
Gschwind et al. 2015	+	?	+	+	+	?
Htut et al. 2018	+	+	+	+	?	?
Hughes et al. 2014	?	?	+	?	?	?
Imam et al. 2017	+	?	+	+	?	?
Jorgensen et al. 2013	+	−	+	+	?	−
Jung et al. 2015	?	?	+	?	?	?
Karahan et al. 2015	+	?	+	?	?	?
Ku et al. 2019	+	?	+	+	?	?
Kwok & Pua 2016	+	?	+	+	+	?
Lauze et al. 2017	+	−	+	?	?	−
Lauze et al. 2018	+	−	+	?	?	−
Laver et al. 2012	+	+	+	+	?	?
Lee et al. 2013	?	?	+	?	?	?
Lee et al. 2015	+	−	+	+	?	−
Lee et al. 2017	+	?	+	+	?	?
Liao et al. 2019	+	?	+	+	?	?
Liao, Chen & Wang 2019	+	+	+	+	?	?
Lim et al. 2017	?	?	+	?	?	?
Lin et al. 2007	?	?	+	?	?	?
Maillot et al. 2012	?	?	+	?	?	?
Maillot et al. 2014	+	?	+	+	?	?
Martel et al. 2018	?	?	+	?	?	?
Micarelli et al. 2019	?	+	+	?	?	?
Mirelman et al. 2016	+	+	+	+	+	+
MonteiroJunior et al.2017	+	+	+	?	?	?
Montero-Alia et al. 2019	−	−	−	?	+	−
Morat et al. 2019	+	?	+	?	?	?
Padala et al. 2012	?	?	+	?	?	?
Park et al. 2015	?	?	+	?	?	?
Pichierri et al. 2012	+	−	?	+	?	−
Pitta et al. 2019	+	?	+	+	?	?
Pluchino et al. 2012	+	−	+	?	?	−
Ray et al. 2012	−	−	−	?	?	−
Rendon et al. 2012	?	?	+	+	?	?
Rutkowski et al. 2019	−	−	−	?	+	−
Sajid et al. 2016	−	?	+	+	−	−
Santamaria et al. 2018	?	?	+	?	?	?
Santos et al. 2019	?	−	+	?	?	−
Sato et al. 2015	+	?	+	+	?	?
Schoene et al. 2013	+	?	+	+	?	?
Schwenk et al. 2014	+	+	+	+	?	?
Schättin et al. 2016	+	+	+	+	?	?
Segura-Orti et al. 2019	+	?	+	+	−	−
Singh et al. 2013	?	?	+	+	?	?
Smaerup et al. 2015	+	?	+	?	−	−
Smaerup et al. 2016	+	?	+	?	?	?
Stanmore et al. 2019	?	+	+	?	?	?
Sutanto et al. 2019	?	?	+	?	+	?
Szturm et al. 2011	?	+	+	+	?	?
Tollar et al. 2019	?	+	+	+	?	?
Toulotte et al. 2012	+	+	+	?	?	?
Tsang & Fu 2016	?	?	+	?	?	?
Uzor & Baillie 2019	+	−	−	+	?	−
Villumsen et al. 2019	+	?	+	+	+	?
Yesilyaprak et al. 2016	?	?	+	+	?	?
Yuen et al. 2019	?	+	+	+	?	?

Figure C2. Summary of risk of bias across RCTs (n=58) included in meta-analysis.

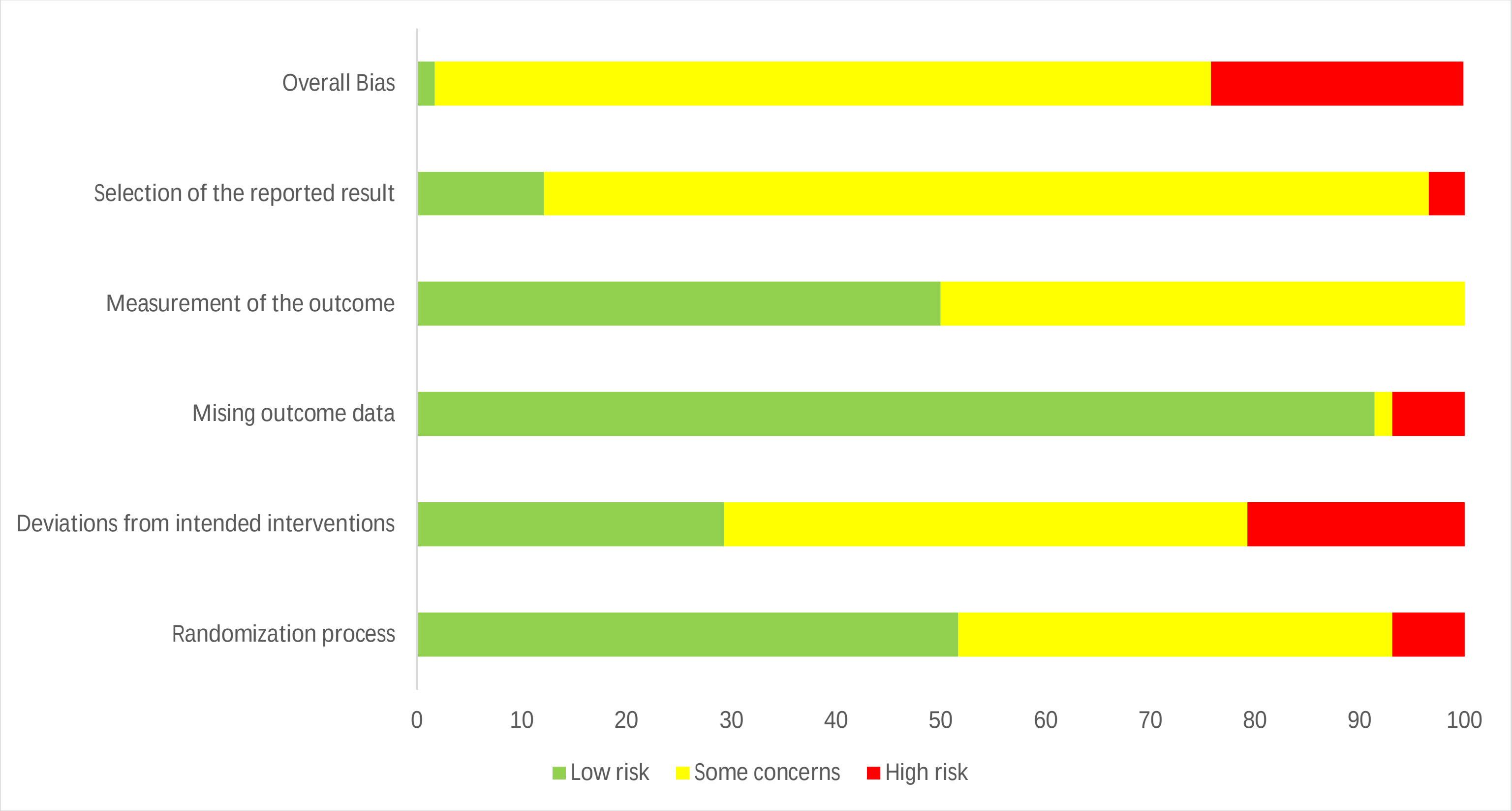

Table C3. Summary of Findings

Exergaming intervention compared to comparison group intervention[3]

Patients or population: Older adults aged 60 years or older with no common neurological condition
Settings: Varies
Intervention: Exergaming
Comparison: Other exercising protocol (other exergaming, other exercising, cognitive training) and no exercising protocol (control), i.e. active and inactive control respectively

Outcomes	SMD with 95% Confidence Interval<br><br>Heterogeneity (I^2, p)	No of studies with high overall risk of bias[2]	No of Participants (Studies)	Publication bias[5]	Certainty of the evidence (GRADE)	Comments
Walking post intervention	-0.21 [-0.36, -0.06]<br><br>76.3%, <.0001	14	3102 (58)	Not serious	⊕⊕⊕◯Moderate[1]	Walking was assessed with validated and standardized meters (Timed Up & Go test, walking speed test, 2- and 6-minute walking test, Dynamic Gait Index, Functional Gait Assessment, Tinetti's Gait).
Walking post follow-up	-0.32 [-0.64, 0.00]<br><br>72.8%, <.0001	4	1028 (13)	Not serious	⊕⊕◯◯Low[14]	Walking was assessed with validated and standardized meters (Timed Up & Go test, walking speed test, 2-minute walking test, Functional Gait Assessment and Tinetti's Gait).

[1] Downgrading by one level due to inconsistency: Substantial heterogeneity.

[2] No downgrading due to risk of bias, as meta-regression analysis did not revealed significant association when high risk of bias was assessed by domains. In studies post intervention, high risk of bias was identified in randomization process (4), deviations from intended intervention (12), missing outcome data (4) and selection of the reported results (2). In studies post follow-up, high risk of bias was identified in randomization process (1), deviations from intended intervention (4) and missing outcome data (1).

[3] No downgrading due to indirectness; Correspondence to review's PICO criteria.

[4] Downgrading by one level due to imprecision: Wide confidence interval including null effect and small amount of studies.

[5] No downgrading due to publication bias: Asymmetry in the funnel plots indicated possibility of publication bias, but studies in favor of both groups has been published equally.

REFERENCES

1. Bacha J, Gomes G, De Freitas T, et al. Effects of kinect adventures games versus conventional physical therapy on postural control in elderly people: A randomized controlled trial. *Games Health J*. 2018;7:24-36. doi:10.1089/g4h.2017.0065

2. Bieryla KA, Dold NM. Feasibility of Wii Fit training to improve clinical measures of balance in older adults. *Clin Interv Aging*. 2013;8:775-781. doi:10.2147/CIA.S46164

3. Bieryla KA. Xbox Kinect training to improve clinical measures of balance in older adults: A pilot study. *Aging Clin Exp Res*. 2016;28:451-457. doi:10.1007/s40520-015-0452-y

4. Chow DHK, Mann SKF. Effect of cyber-golfing on balance amongst the elderly in Hong Kong: A pilot randomised trial. *Hong Kong J Occup Ther*. 2015;26:9-13. doi:10.1016/j.hkjot.2015.08.001

5. Christiansen CL, Bade MJ, Davidson BS, Dayton MR, Stevens-Lapsley JE. Effects of weight-bearing biofeedback training on functional movement patterns following total knee arthroplasty: A randomized controlled trial. *J Orthop Sports Phys Ther*. 2015;45:647-655. doi:10.2519/jospt.2015.5593

6. Daniel K. Wii-hab for pre-frail older adults. *Rehabil Nurs*. 2012;37:195-201. doi:10.1002/rnj.25

7. Delbroek T, Vermeylen W, Spildooren J. The effect of cognitive-motor dual task training with the biorescue force platform on cognition, balance and dual task performance in institutionalized older adults: a randomized controlled trial. *J Phys Ther Sci*. 2017;29:1137-1143. doi:10.1589/jpts.29.1137

8. Eggenberger P, Theill N, Holenstein S, Schumacher V, de Bruin ED. Multicomponent physical exercise with simultaneous cognitive training to enhance dual-task walking of older adults: A secondary analysis of a 6-month randomized controlled trial with I-year follow-up. *Clin Interv Aging*. 2015;10:1711-1732. doi:10.2147/CIA.S91997

9. Fung V, Ho A, Shaffer J, Chung E, Gomez M. Use of Nintendo Wii FitTM in the rehabilitation of outpatients following total knee replacement: A preliminary randomised controlled trial. *Physiotherapy*. 2012;98:183-188. doi:10.1016/j.physio.2012.04.001

10. Gomes GCV, Simoes M do S, Lin SM, et al. Feasibility, safety, acceptability, and functional outcomes of playing Nintendo Wii Fit PlusTM for frail older adults: A randomized feasibility clinical trial. *Maturitas*. 2018;118:20-28. doi:10.1016/j.maturitas.2018.10.002

11. Gschwind YJ, Eichberg S, Ejupi A, et al. ICT-based system to predict and prevent falls (iStoppFalls): Results from an international multicenter randomized controlled trial. *Eur Rev Aging Phys Act*. 2015;12:1-11. doi:10.1186/s11556-015-0155-6

12. Htut TZC, Hiengkaew V, Jalayondeja C, Vongsirinavarat M. Effects of physical, virtual reality-based, and brain exercise on physical, cognition, and preference in older persons: A randomized controlled trial. *Eur Rev Aging Phys Act*. 2018;15:1-12. doi:10.1186/s11556-018-0199-5

13. Hughes TF, Flatt JD, Fu B, Butters MA, Chang CCH, Ganguli M. Interactive video gaming compared with health education in older adults with mild cognitive impairment: A feasibility study. *Int J Geriatr Psychiatry*. 2014;29:890-898. doi:10.1002/gps.4075

14. Imam B, Miller WC, Finlayson H, Eng JJ, Jarus T. A randomized controlled trial to evaluate the feasibility of the Wii Fit for improving walking in older adults with lower limb amputation. *Clin Rehabil*. 2017;31:82-92. doi:10.1177/0269215515623601

15. Jorgensen MG, Laessoe U, Hendriksen C, Nielsen OBF, Aagaard P. Efficacy of nintendo wii training on mechanical leg muscle function and postural balance in community-dwelling older adults: A randomized controlled trial. *Journals Gerontol - Ser A Biol Sci Med Sci*. 2013;68:845-852. doi:10.1093/gerona/gls222

16. Jung DI, Ko DS, Jeong MA. Kinematic effect of Nintendo Wii™ sports program exercise on obstacle gait in elderly women with falling risk. *J Phys Ther Sci*. 2015;27:1397-1400. doi:10.1589/jpts.27.1397

17. Karahan AY, Tok F, Taşkın H, Küçüksaraç S, Başaran A, Yildirim P. Effects of exergames on balance, functional mobility, and quality of life of geriatrics versus home exercise programme: Randomized controlled study. *Cent Eur J Public Health*. 2015;23:S14-S18. doi:10.21101/cejph.a4081

18. Ku J, Kim YJ, Cho S, Lim T, Lee HS, Kang YJ. Three-dimensional augmented reality system for balance and mobility rehabilitation in the elderly: A randomized controlled trial. *Cyberpsychology, Behav Soc Netw*. 2019;22:132-141. doi:10.1089/cyber.2018.0261

19. Kwok BC, Pua YH. Effects of Wii Active exercises on fear of falling and functional outcomes in community-dwelling older adults: A randomised control trial. *Age Ageing*. 2016;45:621-627. doi:10.1093/ageing/afw108

20. Lauzé M, Martel D, Aubertin-Leheudre M. Feasibility and effects of a physical activity program using gerontechnology in assisted living communities for Older Adults. *J Am Med Dir Assoc*. 2017;18:1069-1075. doi:10.1016/j.jamda.2017.06.030

21. Lauzé M, Martel D, Agnoux A, et al. Feasibility, acceptability and effects of a home-based exercise program using a gerontechnology on physical capacities after a minor injury in community-living older adults: A pilot study. *J Nutr Heal Aging*. 2018;22:16-25. doi:10.1007/s12603-017-0938-8

22. Laver K, George S, Ratcliffe J, et al. Use of an interactive video gaming program compared with conventional physiotherapy for hospitalised older adults: A feasibility trial. *Disabil Rehabil*. 2012;34:1802-1808. doi:10.3109/09638288.2012.662570

23. Lee S, Shin S. Effectiveness of virtual reality using video gaming technology in elderly adults with diabetes mellitus. *Diabetes Technol Ther*. 2013;15:489-496. doi:10.1089/dia.2013.0050

24. Lee M, Son J, Kim J, Yoon BC. Individualized feedback-based virtual reality exercise improves older women's self-perceived health: A randomized controlled trial. *Arch Gerontol Geriatr*. 2015;61:154-160. doi:10.1016/j.archger.2015.06.010

25. Lee Y, Choi W, Lee K, Song C, Lee S. Virtual reality training with three-dimensional video games improves postural balance and lower extremity strength in community-dwelling older adults. *J Aging Phys Act*. 2017;25:621-627. doi:10.1123/japa.2015-0271

26. Liao Y-Y, Chen I-H, Lin Y-J, Chen Y, Hsu W-C. Effects of virtual reality-based physical and cognitive training on executive function and dual-task gait performance in older adults with mild cognitive impairment: A randomized control trial. *Front Aging Neurosci*. 2019;11:1-10. doi:10.3389/fnagi.2019.00162

27. Liao Y-Y, Chen I-H, Wang R-Y. Effects of Kinect-based exergaming on frailty status and physical performance in prefrail and frail elderly: A randomized controlled trial. *Sci Rep*. 2019;9:1-9. doi:10.1038/s41598-019-45767-y

28. Lim J, Cho JJ, Kim J, Kim Y, Yoon BC. Design of virtual reality training program for prevention of falling in the elderly: A pilot study on complex versus balance exercises. *Eur J Integr Med*. 2017;15:64-67. doi:10.1016/j.eujim.2017.09.008

29. Lin DH, Lin YF, Chai HM, Han YC, Jan MH. Comparison of proprioceptive functions between computerized proprioception facilitation exercise and closed kinetic chain exercise in patients with knee osteoarthritis. *Clin Rheumatol*. 2007;26:520-528. doi:10.1007/s10067-006-0324-0

30. Maillot P, Perrot A, Hartley A. Effects of interactive physical-activity video-game training on physical and cognitive function in older adults. *Psychol Aging*. 2012;27:589-600. doi:10.1037/a0026268

31. Maillot P, Perrot A, Hartley A, Do MC. The braking force in walking: Age-related differences and improvement in older adults with exergame training. *J Aging Phys Act*. 2014;22:518-526. doi:10.1123/JAPA.2013-0001

32. Martel D, Lauzé M, Agnoux A, et al. Comparing the effects of a home-based exercise program using a gerontechnology to a community-based group exercise program on functional capacities in older adults after a minor injury. *Exp Gerontol*. 2018;108:41-47. doi:10.1016/j.exger.2018.03.016

33. Micarelli A, Viziano A, Micarelli B, Augimeri I, Alessandrini M. Vestibular rehabilitation in older adults with and without mild cognitive impairment: Effects of virtual reality using a head-mounted display. *Arch Gerontol Geriatr*. 2019;83:246-256. doi:10.1016/j.archger.2019.05.008

34. Mirelman A, Rochester L, Maidan I, et al. Addition of a non-immersive virtual reality component to treadmill training to reduce fall risk in older adults (V-TIME): A randomised controlled trial. *Lancet*. 2016;388:1170-1182. doi:10.1016/S0140-6736(16)31325-3

35. Monteiro-Junior RS, Figueiredo LFDS, Maciel-Pinheiro PDT, et al. Virtual reality–based physical exercise with exergames (PhysEx) improves mental and physical health of institutionalized older adults. *J Am Med Dir Assoc*. 2017;18:454.e1-454.e9. doi:10.1016/j.jamda.2017.01.001

36. Montero-Alía P, Miralles-Basseda R, López-Jiménez T, et al. Controlled trial of balance training using a video game console in community-dwelling older adults. *Age Ageing*. 2019;48:506-512. doi:10.1093/ageing/afz047

37. Morat M, Bakker J, Hammes V, et al. Effects of stepping exergames under stable versus unstable conditions on balance and strength in healthy community-dwelling older adults: A three-armed randomized controlled trial. *Exp Gerontol*. 2019;127:110719. doi:10.1016/j.exger.2019.110719

38. Padala KP, Padala PR, Malloy TR, et al. Wii-fit for improving gait and balance in an assisted living facility: A pilot study. *J Aging Res*. 2012;2012. doi:10.1155/2012/597573

39. Park EC, Kim SG, Lee CW. The effects of virtual reality game exercise on balance and gait of the elderly. *J Phys Ther Sci*. 2015;27:1157-1159. doi:10.1589/jpts.27.1157

40. Pichierri G, Murer K, de Bruin ED. A cognitive-motor intervention using a dance video game to enhance foot placement accuracy and gait under dual task conditions in older adults: A randomized controlled trial. *BMC Geriatr*. 2012;12:74. doi:10.1186/1471-2318-12-74

41. Pitta A, Pereira G, Lara JPR, et al. The Effects of Different Exergame Intensity Training on Walking Speed in Older Women. *Games Health J*. 2020;9:121-128. doi:10.1089/g4h.2019.0109

42. Santos GOR, Wolf R, Silva MM, Rodacki ALF, Pereira G. Does exercise intensity increment in exergame promote changes in strength, functional capacity and perceptual parameters in pre-frail older women? A randomized controlled trial. *Exp Gerontol*. 2019;116:25-30. doi:10.1016/j.exger.2018.12.009

43. Pluchino A, Lee SY, Asfour S, Roos BA, Signorile JF. Pilot study comparing changes in postural control after training using a video game balance board program and 2 standard activity-based balance intervention programs. *Arch Phys Med Rehabil*. 2012;93:1138-1146. doi:10.1016/j.apmr.2012.01.023

44. Ray C, Melton F, Ramirez R, Keller D. The effects of a 15-week exercise intervention on fitness and postural control in older adults. *Act Adapt Aging*. 2012;36:227-241. doi:10.1080/01924788.2012.696236

45. Rendon AA, Lohman EB, Thorpe D, Johnson EG, Medina E, Bradley B. The effect of virtual reality gaming on dynamic balance in older adults. *Age Ageing*. 2012;41:549-552. doi:10.1093/ageing/afs053

46. Rutkowski S, Rutkowska A, Jastrzebski D, Racheniuk H, Pawelczyk W, Szczegielniak J. Effect of virtual reality-based rehabilitation on physical fitness in patients with chronic obstructive pulmonary disease. *J Hum Kinet*. 2019;69:149-157. doi:10.2478/hukin-2019-0022

47. Sajid S, Dale W, Mustian K, et al. Novel physical activity interventions for older patients with prostate cancer on hormone therapy: A pilot randomized study. *J Geriatr Oncol*. 2016;7:71-80. doi:10.1016/j.jgo.2016.02.002

48. Santamaría KG, Fonseca AS, Jiménez JM, Mora LCS. Balance, attention and concentration improvements following an exergame training program in elderly. *Retos-Nuevas Tendencias En Educ Fis Deport Y Recreacion*. 2018;2041:102-105.

49. Sato K, Kuroki K, Saiki S, Nagatomi R. Improving walking, muscle strength, and balance in the elderly with an exergame using kinect: A randomized controlled trial. *Games Health J*. 2015;4:161-167. doi:10.1089/g4h.2014.0057

50. Schoene D, Lord SR, Delbaere K, Severino C, Davies TA, Smith ST. A randomized controlled pilot study of home-based step training in older people using videogame technology. *PLoS One*. 2013;8:e57734-e57734. doi:10.1371/journal.pone.0057734

51. Schwenk M, Grewal GS, Honarvar B, et al. Interactive balance training integrating sensor-based visual feedback of movement performance: A pilot study in older adults. *J*

Neuroeng Rehabil. 2014;11:164. doi:10.1186/1743-0003-11-164

52. Schättin A, Arner R, Gennaro F, de Bruin ED. Adaptations of prefrontal brain activity, executive functions, and gait in healthy elderly following exergame and balance training: A randomized-controlled study. *Front Aging Neurosci*. 2016;8. doi:10.3389/fnagi.2016.00278

53. Segura-Ortí E, Pérez-Domínguez B, Ortega-Pérez de Villar L, et al. Virtual reality exercise intradialysis to improve physical function: A feasibility randomized trial. *Scand J Med Sci Sport*. 2019;29:89-94. doi:10.1111/sms.13304

54. Singh DKA, Rajaratnam BS, Palaniswamy V, Raman VP, Bong PS, Pearson H. Effects of balance-focused interactive games compared to therapeutic balance classes for older women. *Climacteric*. 2013;16:141-146. doi:10.3109/13697137.2012.664832

55. Smaerup M, Grönvall E, Larsen SB, Laessoe U, Henriksen J-J, Damsgaard EM. Computer-assisted training as a complement in rehabilitation of patients with chronic vestibular dizziness: A randomized controlled trial. *Arch Phys Med Rehabil*. 2015;96:395-401. doi:10.1016/j.apmr.2014.10.005

56. Smaerup M, Laessoe U, Grönvall E, Henriksen JJ, Damsgaard EM. The use of computer-assisted home exercises to preserve physical function after a vestibular rehabilitation program: A randomized controlled study. *Rehabil Res Pract*. 2016;2016. doi:10.1155/2016/7026317

57. Stanmore EK, Mavroeidi A, de Jong LD, et al. The effectiveness and cost-effectiveness of strength and balance Exergames to reduce falls risk for people aged 55 years and older in UK assisted living facilities: A multi-centre, cluster randomised controlled trial. *BMC Med*. 2019;17:49. doi:10.1186/s12916-019-1278-9

58. Sutanto YS, Makhabah DN, Aphridasari J, Doewes M, Suradi AN. Videogame assisted exercise training in patients with chronic obstructive pulmonary disease: A preliminary study. *Pulmonology*. 2019;25:275-282. doi:10.1016/j.pulmoe.2019.03.007

59. Szturm T, Betker AL, Moussavi Z, Desai A, Goodman V. Effects of an interactive computer game exercise regimen on balance impairment in frail community-dwelling older adults: A randomized controlled trial. *Phys Ther*. 2011;91:1449-1462. doi:10.2522/ptj.20090205

60. Tollar J, Nagy F, Moizs M, Toth BE, Sanders LMJ, Hortobagyi T. Diverse exercises similarly reduce older adults' mobility limitations. *Med Sci Sports Exerc*. 2019;51:1809-1816. doi:10.1249/MSS.0000000000002001

61. Toulotte C, Toursel C, Olivier N. Wii Fit (R) training vs. adapted physical activities: Which one is the most appropriate to improve the balance of independent senior subjects? A randomized controlled study. *Clin Rehabil*. 2012;26:827-835. doi:10.1177/0269215511434996

62. Tsang WWN, Fu ASN. Virtual reality exercise to improve balance control in older adults at risk of falling. *Hong Kong Med J*. 2016;22:S19-S22.

63. Uzor S, Baillie L. Recov-R: Evaluation of a home-based tailored exergame system to reduce fall risk in seniors. *Acm Trans Comput Interact*. 2019;26. doi:10.1145/3325280

64. Villumsen BR, Jorgensen MG, Frystyk J, Hørdam B, Borre M. Home-based 'exergaming' was safe and significantly improved 6-min walking distance in patients

with prostate cancer: A single-blinded randomised controlled trial. *BJU Int*. 2019;124:600-608. doi:10.1111/bju.14782

65. Yeşilyaprak SS, Yildirim MŞ, Tomruk M, Ertekin Ö, Algun ZC. Comparison of the effects of virtual reality-based balance exercises and conventional exercises on balance and fall risk in older adults living in nursing homes in Turkey. *Physiother Theory Pract*. 2016;32:191-201. doi:10.3109/09593985.2015.1138009

66. Yuen HK, Lowman JD, Oster RA, de Andrade JA. Home-based pulmonary rehabilitation for patients with idiopathic pulmonary fibrosis: A pilot study. *J Cardiopulm Rehabil Prev*. 2019;39:281-284. doi:10.1097/HCR.0000000000000418
